# Ni- and Zn-Doping Effects on Cu/SiO_2_ Catalysts in Nonoxidative Ethanol Dehydrogenation

**DOI:** 10.1021/acs.iecr.5c04241

**Published:** 2026-02-24

**Authors:** Tomas Pokorny, Petr Machac, Zdenek Moravec, Lucie Simonikova, Lucie Leonova, Zuzana Hlavenkova, David Skoda, Katerina Pacultova, Katerina Karaskova, Ales Styskalik

**Affiliations:** † Department of Chemistry, 37748 Masaryk University, Kotlarska 2, CZ-61137 Brno, Czech Republic; ‡ CEITEC, Masaryk University, Kamenice 5, CZ-62500 Brno, Czech Republic; § Centre of Polymer Systems, 48362 Tomas Bata University in Zlín, Tr. T. Bati 5678, CZ-76001 Zlín, Czech Republic; ∥ Institute of Environmental Technology, CEET, VSB-TUO, 17. listopadu 2172/15, CZ-70800 Ostrava, Czech Republic

## Abstract

Nonoxidative ethanol dehydrogenation opens a pathway for the sustainable production of acetaldehyde and butadiene. One crucial aspect of producing butadiene by the Lebedev process is the high-temperature stability of ethanol to acetaldehyde conversion. However, copper-based catalysts, despite exhibiting high activity and selectivity, suffer from sintering and coking and need to be improved for successful industrial applications. Herein, we show Cu-based (∼2.5 wt %) catalysts doped with Ni and Zn (0.028–0.36 wt %) to improve the catalytic performance of nanoparticles. The catalysts were prepared by hydrolytic sol–gel and dry impregnation methods. STEM analysis determined the nanoparticle sizes in the 1.9–2.8 nm range. Ni-doped catalysts outperformed the parent Cu catalysts in ethanol dehydrogenation activity at lower temperatures (185–220 °C) but suffered from faster deactivation. The Zn-doped catalysts exhibited improved high-temperature stability. For these materials, acetaldehyde selectivity fluctuated around ∼90% and acetaldehyde productivity reached 3.63 g g^–1^ h^–1^ at 290 °C and a WHSV of 4.73 h^–1^. The improved stability of the Zn-doped samples was correlated with lower coke formation (XPS, TG analysis, and Raman spectroscopy).

## Graphical abstract

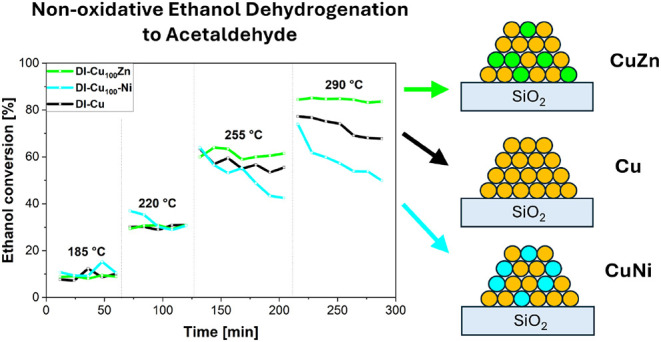

## Introduction

The depletion of finite petroleum reserves, coupled with the urgent need to address environmental concerns associated with fossil fuel consumption, has compelled the global community to seek cleaner and sustainable sources.^1–3^ Bioethanol, produced by sugar fermentation,^4^ has emerged as one of the promising alternatives in the transition away from petroleum-based products.^5^

Acetaldehyde can be produced by the bioethanol conversion.^6,7^ It is an essential resource in the production chain of acetic acid, ethyl acetate, pentaerythritol, pyridine bases, and polymers.^8–10^ The Wacker process is the current method for producing acetaldehyde, involving the oxidation of ethylene to acetaldehyde over a homogeneous catalyst (PdCl_2_ and CuCl_2_).^11^ Notably, ethylene production by steam cracking has attracted attention due to its tremendous CO_2_ production.^12^

Nonoxidative ethanol dehydrogenation offers a potential key for sustainable acetaldehyde production, a high atom economy, and hydrogen as a side product (eq 1).^13^ Ethanol dehydrogenation is an endothermic process.^14,15^ Thus, performing dehydrogenation at higher temperatures allows us to apply shorter contact times and leads to better acetaldehyde productivities. Furthermore, the conversion of ethanol to acetaldehyde is the first step in the Lebedev process,^16,17^ intended for the sustainable production of butadiene. The ethanol-to-butadiene process requires temperatures between 300 and 400 °C.^17^ Therefore, it is necessary to develop stable catalysts capable of withstanding higher temperatures. The catalysts also need to be highly selective because other reactions might be promoted, such as ethanol dehydration (eq 2 and 3) or decomposition (eq 4).^18–20^

1 $${\mathrm{CH}}_{3}{\mathrm{CH}}_{2}\mathrm{OH}\to {\mathrm{CH}}_{3}\mathrm{CHO}+H_{2}$$

2 $$2{\mathrm{CH}}_{3}{\mathrm{CH}}_{2}\mathrm{OH}\to \left({\mathrm{CH}}_{3}{\mathrm{CH}}_{2}\right)O+H_{2}O$$

3 $${\mathrm{CH}}_{3}{\mathrm{CH}}_{2}\mathrm{OH}\to H_{2}C\text{=}{\mathrm{CH}}_{2}+H_{2}O$$

4 $${\mathrm{CH}}_{3}{\mathrm{CH}}_{2}\mathrm{OH}\to {\mathrm{CH}}_{4}+\mathrm{CO}+H_{2}$$

Many experimental studies on nonoxidative ethanol dehydrogenation have highlighted copper as the most active and selective catalyst.^21–23^ Unfortunately, it has been reported that copper nanoparticle sintering leads to catalyst deactivation during the catalytic reaction.^6,22,24–26^ The propensity of Cu NPs to sinter is caused by their low Tammann temperature (405 °C).^14,27^ Several methods have been studied to suppress Cu particle migration and sintering: (i) formation of mechanical barriers (i.e., Cu particle encapsulation in SiO_2_,^14^ particles of metals and metal oxides as mechanical barriers^25^), (ii) improving the Cu–support interaction (i.e., addition of ZnO,^27^ formation of copper phyllosilicate phase on the surface^28^), or (iii) alloying Cu with metals exhibiting higher melting points, thus intentionally increasing the Tammann temperature (e.g., Ni).^29^

Amokrane et al. studied the effects of alloying copper microparticles with other transition metals on their catalytic properties in ethanol dehydrogenation. In the case of Ni addition, the conversion improved, but Ni doping negatively affected the selectivity to acetaldehyde (methane production increased). The stability of Cu-based catalysts with time-on-stream has not been improved by Ni doping.^25^ On the contrary, improved stability was reported in a study,^29^ where Ni_0.01_Cu nanoparticles were prepared by the deposition of Cu NPs and subsequential galvanic replacement of Cu for Ni. The improved Cu NP resistance against sintering upon Ni introduction has been suggested as a reason for the better catalytic stability. Ni_0.01_Cu nanoparticles on silica also outperformed monometallic Cu NP catalysts in activity. Similarly, the alloying of active Au with Ni improved the catalytic activity.^30^ Kinetic studies showed in both cases that Ni doping significantly decreased the apparent activation energy of the ethanol dehydrogenation reaction, thereby increasing the catalytic activity.^29,30^

Although better stability against sintering in Ni-doped catalysts is probably caused by their high melting point, ZnO is often used as a catalyst support or dopant for Cu NPs, as it exhibits a strong interaction with both Cu and SiO_2_ ^31,66^ and might work as a mechanical barrier.^14^ For example, Hou et al. reported a highly stable Cu/ZnO@SiO_2_ catalyst (stable at 350 °C for 200 h). The material was prepared from a metal–organic framework containing Cu and Zn via soaking with tetraethoxysilane and subsequent calcination. Its exceptional stability during TOS was ascribed to both SiO_2_ encapsulation and ZnO particles functioning as mechanical barriers for Cu sintering. The ZnO presence also positively influenced the catalytic activity.^14,27^ Finally, Cu^0^/ZnO/ZnAl_2_O_4_ was also used in ethanol dehydrogenation, showing that the nanoparticles were more stable against sintering at higher temperatures (300 and 350 °C) in comparison to the pure Cu catalyst for 6 h on stream. The improved catalysts’ stability has been ascribed to strong metal–support interactions (enhanced by ZnO present in the form of small crystallites).^31^

Coking has been reported as another reason for the Cu-based catalyst deactivation in nonoxidative ethanol dehydrogenation, in addition to Cu particle sintering.^20,25,26,28,32–34^ Advantageously, coked catalysts might be regenerated by oxidative treatment.^28,32–34^ Another possibility is to suppress coking and enhance stability via substoichiometric O_2_ cofeeding directly during the catalytic reaction.^33^ The influence of doping Cu with other elements on coking has been described only scarcely. The Ag–Cu catalyst has been shown to coke extensively and, thus, deactivate rapidly,^25^ and phosphorus doping has been suggested to mitigate coking.^7^ Ni and Zn addition to Cu-based catalysts has not been studied with respect to carbon deposition in nonoxidative ethanol dehydrogenation, to the best of our knowledge.

In this study, we investigate the influence of Ni and Zn doping on the activity, selectivity, and stability of Cu-based catalysts in the dehydrogenation of ethanol to acetaldehyde. Ni and Zn were chosen due to their previously reported enhancement of both activity and stability, though the underlying mechanisms behind these improvements remain incompletely understood. To deepen the insights into this area, we built on our prior work, which explored how copper particle size and preparation method affect the catalytic behavior in nonoxidative ethanol dehydrogenation.^6^ Specifically, the most stable material (Cu/SiO_2_ prepared by the dry impregnation method) and the most active catalyst (Cu/SiO_2_ synthesized by the hydrolytic sol–gel approach) were modified by introducing diluted amounts of Ni and Zn through dry impregnation. We systematically evaluated the impact of these dopants on ethanol conversion, acetaldehyde selectivity, and catalyst stability across a temperature range of 185–325 °C. The long-term stability at high temperatures was tested for the potential application of our materials in the ethanol-to-butadiene process. Comprehensive characterization of both the fresh and spent catalysts was performed to elucidate the origins of the observed differences in the catalytic performance induced by Ni and Zn doping.

## Experimental Section

### General

Cu­(NO_3_)_2_·5/2H_2_O was used as the copper precursor (purchased from Merck). NiCl_2_·6H_2_O was the nickel precursor, and ZnNO_3_·6H_2_O was the zinc precursor for the synthesis (both were from home stocks). Cu, Ni, and Zn were supported on commercial silica Aerosil 300 from Evonik. One sample was prepared by the sol–gel technique from Si­(OC_2_H_5_)_4_ (house stock; purified by vacuum distillation).

The catalysts were prepared by the one-step or two-step dry impregnation applying metal salts on silica supports, denoted as HSG and DI (prepared by using hydrolytic sol–gel with Cu, and dry impregnation on Aerosil 300, respectively).^35^ The samples contained 0.25 and 0.025 wt % nominal Ni loading, respectively. The nominal Cu loading was 2.5 wt %; thus, the Cu:Ni weight ratios were 10 and 100, respectively, and the samples are denoted as **DI-Cu**_**10**_ **Ni** and **DI-Cu**_**100**_ **Ni**. The notation for two-step impregnation is represented by an additional “-“ (e.g., **DI-Cu**_**10**_ **-Ni**), where the order of impregnation is as written in the sample label: copper was impregnated first, and nickel was deposited by the second impregnation. A similar notation was used for the Zn-doped samples. The detailed preparation of each catalyst is described in Supporting Material.

### Characterization

An EMPYREAN instrument (PANalytical) was used to measure the powder X-ray diffraction. The samples were placed on a spinning sample holder.  The Co lamp (λ = 1.78901 Å) was powered at 20 mA and 30 kV. A semiconductor detector was used in the 1D mode. Selected samples were reanalyzed using a Rigaku MiniFlex 600 diffractometer equipped with a CoKα radiation source (λ = 1.7903 Å, 40 kV, 15 mA). A scanning rate of 6°/min and a step of 0.02° were used in order to obtain higher-resolution diffractograms. The resulting data were processed with the Rigaku PDXL2 software. Micrograph surveys of the nanoparticles and elemental mapping were performed by scanning transmission electron microscopy with energy-dispersive X-ray spectroscopy (STEM-EDS) on a Thermo Fisher Scientific Talos F200C instrument. This instrument was equipped with a high-angle annular dark-field (HAADF) STEM detector and a Bruker XFlash 6T|30 EDS detector. The accelerating potential was set to 200 kV. The samples were placed on a copper grid covered with a continuous carbon layer. The particle size distribution was evaluated using the software ImageJ. A minimum of 100 nanoparticles per sample were measured on their longest side. The average particle size was used for the estimation of the number of surface Cu atoms (see Supporting Information).^36^ Thermogravimetry (TG) was performed by a Netzsch STA 449 C Jupiter. The samples were measured in Pt/Rh crucibles. The samples were heated in airflow (100 cm^3^ min^–1^), and the heating rate was 5 °C min^–1^ at 1000 °C. Nitrogen porosimetry was performed by an Autosorb iQ3 (Quantachrome Instruments). The adsorption and desorption isotherms were measured at a temperature of −195.7 °C. The samples were degassed for at least 24 h at 200 °C. The specific surface area was determined by BET analysis from the measured isotherms in the relative pressure range of 0.05 to 0.30. X-ray photoelectron spectroscopy (XPS) was performed on a Kratos Axis Supra instrument equipped with a monochromatic X-ray source with excitation Al K_α_. A binding energy of 284.8 eV for C 1s was used for calibration. Raman spectroscopy was performed on a Nicolet DXR Thermo Raman microscope equipped with a green solid-state diode-pumped laser with an excitation wavelength of 532 nm, a laser power of 7 mW, and an aperture of 50 μm pinhole. The spectra were recorded using a high-resolution grating from 1878 to 50 cm^–1^ under standard ambient conditions. Hydrogen temperature-programmed reduction (H_2_-TPR) was carried out on AutoChem II-2920 equipment (Micromeritics, Atlanta, GA) connected online with a mass spectrometer HPR-20 EGA, Hiden Analytical, software MASsoft (Warrington, England). Before each H_2_-TPR experiment, the sample (0.1 g) was pretreated in Ar (50 cm^3^ min^–1^) at 300 °C for 30 min. The sample was cooled to 50 °C in the same atmosphere and then reduced in a hydrogen–argon mixture (10 mol % H_2_/Ar) at a flow rate of 50 cm^3^ min^–1^ and a constant heating rate of 10 °C min^–1^ up to 700 °C, and held at this temperature for 30 min. The water vapor formed during the TPR measurements was captured in a cold trap. For the evaluation of the amount of reducible species, a correction based on the signal of the neat silica support was used. No detectable release from the monitored mass fragments (*m*/*z* = 14 (N_2_), 18 (H_2_O), 28 (N_2_), 30 (NO), 32 (O_2_), 40 (Ar), and 44 (CO_2_)) was observed; therefore, the hydrogen consumption was determined exclusively from the TCD signals.

### Catalytic Ethanol Dehydrogenation to Acetaldehyde

A fixed-bed catalytic reactor connected to a gas chromatograph was used for the catalytic reaction. Catalytic tests were performed at temperatures of 185, 220, 255, and 290 °C. One temperature step consisted of (i) a heating ramp (5 °C min^–1^) and stabilization at the set temperature (21 min) and (ii) a steady temperature state (60 min at 185 and 220 °C; 84 min at 255 and 290 °C). The analysis of the effluent gas was carried out by an HP 6890 Gas Chromatograph (5 injections at 185 and 220 °C and 7 injections at 255 and 290 °C) equipped with a flame ionization detector (FID) and a Thermo Scientific TG-BOND U column (30 m long, internal diameter of 0.32 mm, film thickness of 10 μm). The stability experiments were carried out for 14 h at 325 °C.^6^ Calcined catalysts (100 mg) were used for the catalytic reaction. All catalysts were adjusted to the same volume with these glass beads (diameter 0.5–1 mm). The void space of the reactor was filled with glass beads. Before the reactions, the catalysts were pretreated in situ by forming gas (5 vol % H_2_ in N_2_, 52.5 cm^3^ min^–1^ total flow) for 2 h at 325 °C to perform the reduction of copper oxides. Pure nitrogen was used as the carrier gas (50 cm^3^ min^–1^) in all catalytic reactions; ethanol was fed by an NE-300 syringe pump with a WHSV of 4.73 h^–1^ (7.11 mol % of ethanol in N_2_). Pentane (5 mol % in ethanol feed) was used as the internal standard. The tests were carried out at atmospheric pressure. The catalytic data were used to estimate the apparent activation energy (*E*_a_), as described in the Supporting Information.

## Results and Discussion

### Characterization

Recently, we have compared four synthetic techniques for the preparation of Cu/SiO_2_ catalysts:^6^ dry impregnation (DI-Cu), hydrolytic sol–gel (HSG-Cu), solvothermal hot injection, followed by Cu NP deposition on silica (SHI) and strong electrostatic adsorption (SEA). The highest activity in ethanol dehydrogenation was exhibited by HSG-Cu (the smallest average particle size, 1.3 nm), while DI-Cu on Aerosil 300 support showed the best stability during the time-on-stream (TOS; 3.4 nm average particle size via STEM; crystallite size up to 32 nm according to XRD). Still, the samples deactivated relatively rapidly.^6^ Here, we have chosen the preparation of the most stable catalysts (dry impregnation)^6^ and applied it to study the metal doping effect on catalytic activity, selectivity, and stability. Cu/SiO_2_ (nominal Cu loading 2.5 wt %) was doped with Ni or Zn (nominal loading 0.25 and 0.025 wt %). The catalysts were prepared by either one-step impregnation or two-step impregnation. The DI preparation method was compared with HSG ( a preparation method providing the most active Cu catalyst),^6^ with Ni deposited by a subsequent impregnation.

The metal loading for all catalysts (Table 1, ICP-OES) varied around the targeted value (2.5 wt %) and was in the range of 1.96–2.61 wt % for copper. Nickel loadings for samples with nominal 0.25 wt % of Ni were in the range of 0.21–0.30 wt % and for samples with nominal 0.025 wt % Ni, in the range of 0.028–0.058 wt %. The experimental zinc loading in the samples with nominal 0.25 wt % Zn was in the range of 0.31–0.36 wt %. Samples with a nominal 0.025 wt % zinc loading exhibited a higher experimental concentration (0.063 wt % Zn) than targeted. The Cu surface concentrations (XPS) of the catalysts prepared by DI were lower than the bulk loadings (0.09–0.62 wt %) with significant variance due to a low signal-to-noise ratio. **HSG-Cu**_**10**_-**Ni** exhibited the highest surface Cu concentration (0.71 wt %). The limit of detection for Ni, Zn, and Cu is ∼0.1 at%.^37^ Therefore, it was not possible to analyze Ni and Zn by XPS.

**Table 1:** Experimental Compositions for All Prepared Catalysts Determined by the ICP-OES Method, and the Surface Cu Content Estimated by XPS

sample	Cu loading^b^ (wt %)	Ni loading^b^ (wt %)	Zn loading^b^ (wt %)	surface Cu content (wt %)	average NP diameter^a^(nm)	standard deviation^a^ (nm)
**HSG-Cu**_**10**_ **-Ni**	1.96	0.300		0.71	2.1	0.6
**DI-Cu**_**10**_ **Ni**	1.98	0.210		0.12	2.3	0.6
**DI-Cu**_**100**_ **Ni**	2.12	0.028		0.09	2.1	0.5
**DI-Cu**_**10**_ **-Ni**	2.19	0.230		0.46	2.3	1.2
**DI-Cu**_**100**_ **-Ni**	2.33	0.058		0.37	2.0	0.5
**DI-Ni-Cu**_**100**_	2.21	0.032		0.24	2.2	0.7
**DI-Cu**_**10**_ **Zn**	2.30		0.310	0.31	2.1	0.4
**DI-Cu**_**100**_ **Zn**	2.61		0.063	0.62	2.6	0.7
**DI-Cu**_**10**_ **-Zn**	2.20		0.360	0.61	2.1	0.4

a Metal loadings were determined by ICP-OES with a standard deviation of 0.001 wt %.

b The nanoparticle size distribution and standard deviation by the graphic analysis of the micrograph surveys were collected by STEM analyses.

The surfaces of the samples were further analyzed by using the XPS method (Figures 1 and S1–S8). The Si 2p spectra of all fresh-calcined and spent catalysts displayed the same peak at 103.7 eV, corresponding to SiO_2_.^38^ Similarly, the O 1s peak originated mostly from SiO_2_ (533 eV).^39^ Also, the C 1s spectra of the fresh-calcined samples did not significantly differ and showed the expected presence of adventitious carbon.^40^ The O 1s, Si 2p, and C 1s spectra are displayed in the Supporting Information (Figures S1–S8). The Cu 2p spectra confirmed the presence of oxidized Cu^2+^ species (peak at 935.3 eV and satellite peak at ∼943 eV) for all fresh-calcined catalysts (calcined in air; Figure 1 left, Figures S1 and S2).^41^ All spent catalysts (Figure 1 right, Figures S1 and S2) were reduced after the catalytic cycle, and the Cu 2p spectra exhibited a peak at 932.7 eV belonging to Cu^0^ or Cu^+^ (indistinguishable by XPS).^42^ Neither nickel nor zinc doping influenced the Cu 2p binding energies, probably due to their low content in the samples (Figure S9).

**Figure 1:**
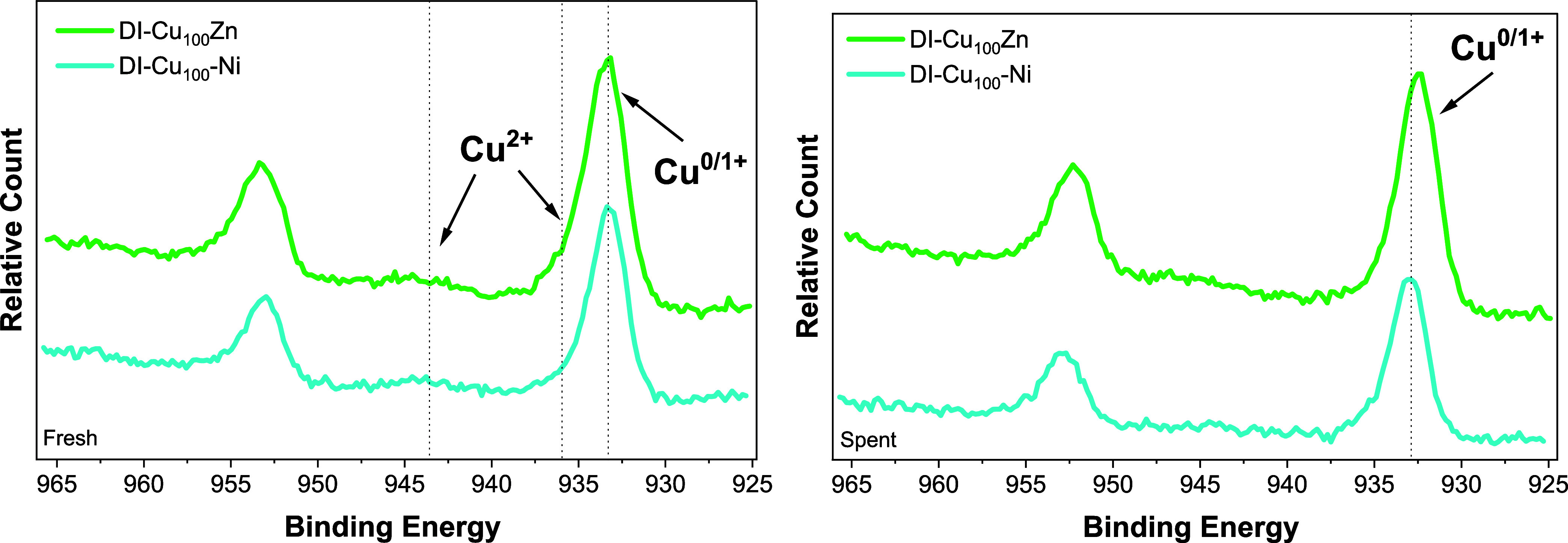
Cu 2p spectra of the chosen Ni- and Zn-doped catalysts (fresh-calcined: left, spent: right).

The samples prepared by dry impregnation were deposited on a commercial silica support (Aerosil 300, 284 m^2^ g^–1^, 1.55 cm^3^ g^–1^, average pore size 10.8 nm; isotherm shown in Figure S10). The trends in porosity indicate a decrease of the specific surface area and pore volume after impregnation and calcination (Table S1). The specific surface area of the catalysts prepared by one-step impregnation was 261–326 m^2^ g^–1^, the pore volume was 1.06–1.85 cm^3^ g^–1^, and the average pore size was 16.2–22.8 nm. Apparently, a more significant effect on porosity was usually observed with the two-step impregnation than with the one-step. The surface specific areas of these samples (i.e., **DI-Cu**_**10**_ **-Ni**, **DI-Cu**_**100**_ **-Ni**, **DI–Ni-Cu**_**100**_, and **DI-Cu**_**10**_ **-Zn**) varied from 219 to 278 m^2^ g^–1^, pore volumes from 0.43 to 1.20 cm^3^ g^–1^, and average pore sizes from 7.9 to 22.8 nm. The samples prepared by one-step impregnation exhibited higher SA_BET_ and *V*_total_ values in most of the cases. Finally, the specific surface area of the **HSG-Cu**_**10**_ **-Ni** catalyst was 485 m^2^ g^–1^, pore volume was 0.61 cm^3^ g^–1^, and average pore size was 4.97 nm, slightly higher than that of the parent Cu-based HSG sample (413 m^2^ g^–1^). These numbers, as well as the isotherm shape, confirm the striking difference between the samples deposited on Aerosil 300 (most of the porosity comes from the interparticle voids) and the sample prepared by hydrolytic sol–gel and subsequent Ni impregnation (**HSG-Cu**_**10**_ **-Ni**; mesoporous structure). The isotherms of the fresh-calcined and spent catalysts are shown in Figure S11, and the pore size distributions are displayed in Figure S12.

The catalysts were further characterized by PXRD analysis (Figure S13), and the results were similar to those of the parent Cu-based **DI-Cu** catalyst.^6^ All samples prepared by DI showed diffractions of CuO (ICSD: 98-003-1059)^43^ after thermal treatment in air. On the one hand, the diffraction maxima, d, and lattice parameters were not significantly shifted and did not follow any clear trend (Tables S2 and S3), indicating no observable mixing of CuO with NiO or ZnO in the crystalline CuO particles. On the other hand, no diffractions for separate NiO or ZnO crystallites were observed, probably due to the low Ni and Zn contents or their presence in the amorphous phases. Further PXRD analyses with a lower scanning rate and step did not confirm or disprove the CuO mixing with NiO or ZnO; the changes to d and lattice parameters were negligible with respect to the quality of the diffractograms given by the intrinsically low content of the crystalline phase in the catalysts.

The Debye–Scherrer equation was used to evaluate the CuO crystallite sizes. These were in the 12–28 nm range for all the DI samples. Importantly, the crystallite size must be considered with care. Reanalyses with a lower scanning rate and step suggested that the crystallite sizes were larger by 10 to 20 nm (Figure S13). Considering the large uncertainty in the *L*_*c*_ determination, crystallite sizes between 12 and 28 nm can be treated as effectively constant.

Finally, PXRD analyses confirmed that the calcined **HSG-Cu**_**10**_ **-Ni** remained amorphous after dry impregnation with a Ni precursor, similar to the Cu-based parent HSG catalyst. Noteworthily, the reduction of parent **DI-Cu** and **HSG-Cu** catalysts by H_2_ successfully provided Cu but did not significantly change the crystallite sizes.^6^

STEM analyses were performed on all samples to complement the PXRD data on crystallite sizes and to evaluate the particle size distributions. STEM micrographs of all catalysts after reduction (Figures 2, S14, and S15) show the prevalent presence of small particles with a narrow size distribution (from 2.0 to 2.6 nm), contrary to the PXRD results (see above). The average particle sizes and deviations for all samples are summarized in Table 1. The particle sizes were similar among the samples, and nickel and zinc at various loadings did not significantly affect the particle size. All Ni- and Zn-doped catalysts after reduction showed slightly smaller particles compared to the Cu-based parent **DI-Cu** sample (average particle size 3.4 nm with standard deviation of 0.9 nm).^6^ Insufficient control of homogeneity during dry impregnation synthesis occasionally caused the formation of larger particles (∼20 nm, STEM-EDS, Figure S18) that correlated with the crystallite sizes estimated by the Debye–Scherrer equation from PXRD diffractograms (Figure S13). However, the STEM analyses suggested that these large particles were observed only rarely, while the small XRD-amorphous particles (∼2 nm) were much more abundant.

**Figure 2:**
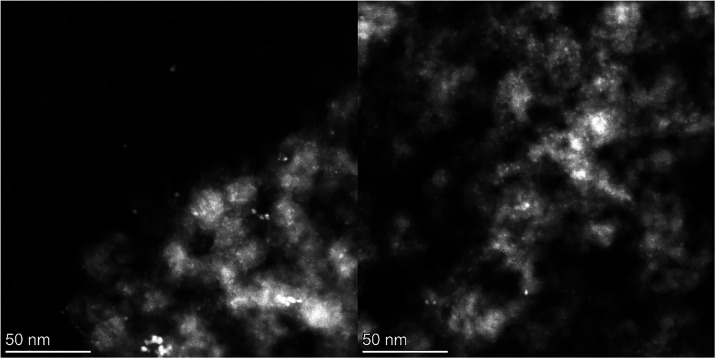
STEM micrograph surveys of the fresh-reduced catalysts (calcined in air followed by H_2_ pretreatment) **DI-Cu**_**10**_ **-Ni** (left) and **DI-Cu**_**10**_ **Zn** (right).

EDS mapping in STEM was used to describe the spatial distribution of Cu, Ni, and Zn in the samples in detail. Extensive STEM-EDS mapping of the Zn-doped samples did not provide any evidence for the formation of separate Zn-rich or ZnO particles. Instead, Cu and Zn were highly homogeneously dispersed over the silica support (Figures 3 and S17). Homogeneous Cu and Ni dispersions were also prevalently observed in the Ni-doped samples (Figures 3 and S16). However, several Ni-rich and crystalline particles (∼20 nm; Cu always present) were observed in **DI-Cu**_**10**_ **-Ni** (Figures 3, S16, and S19). The observed interplanar spacing was consistent with both Cu and Ni (111) interlayer distances (∼2.1 Å).^44,45^ The occasional formation of Ni-rich crystalline particles can have a dramatic impact on the catalytic performance (see the Catalysis and Deactivation sections).

**Figure 3:**
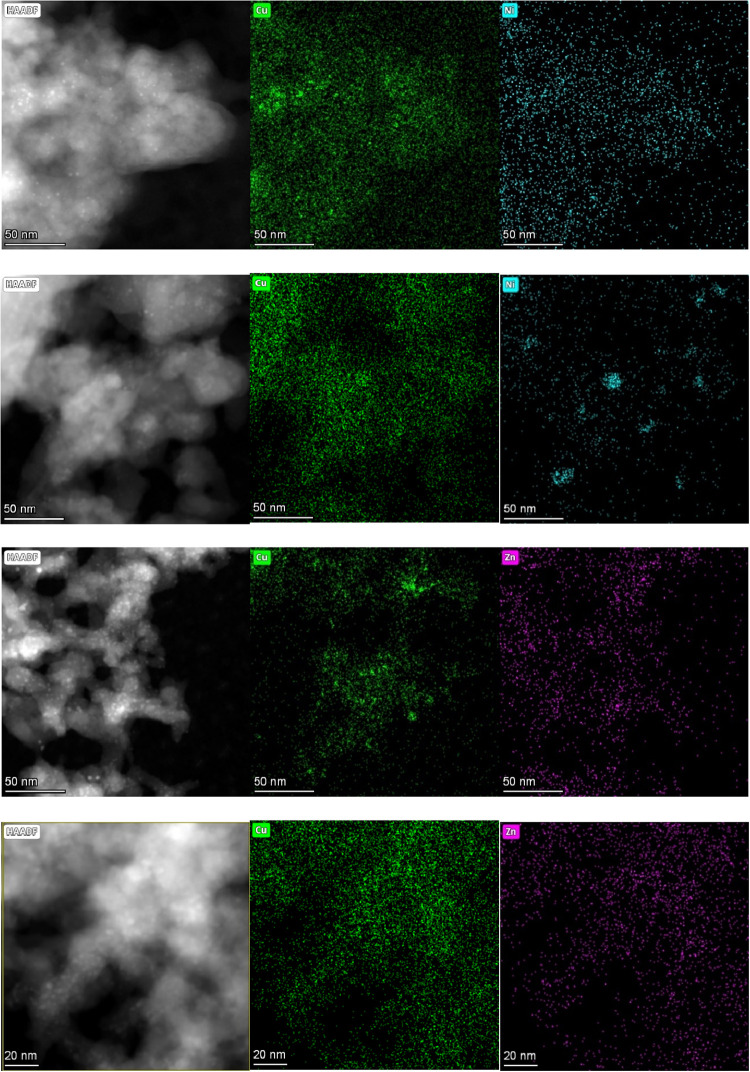
Elemental mapping of fresh-reduced catalysts (calcined in air followed by H_2_ pretreatment) acquired by STEM-EDS analyses (from top to bottom): **DI-Cu**_**10**_ **Ni**, **DI-Cu**_**10**_ **-Ni**, **DI-Cu**_**10**_ **Zn**, and **DI-Cu**_**100**_ **Zn**.

H_2_-TPR analyses were performed to describe the reducibility of the samples in detail. Although apparent reduction degrees of 81–96% were obtained, the metals can be considered fully reduced within the experimental uncertainty associated with low metal loadings; therefore, the discussion of H_2_-TPR analyses is based mainly on the profile shape rather than the absolute H_2_ consumption.

Although the STEM analyses, EDS mapping, PXRD, N_2_-porosimetry, and XPS suggested strong similarities among the parent **DI-Cu** material and Ni- and Zn-doped samples, the H_2_-TPR profiles revealed significant differences apparently resulting from Ni and Zn doping (Figures 4 and 5). The **DI-Cu** sample exhibited one broad peak with a maximum at ∼240 °C and a shoulder at ∼255 °C. These temperatures are in good agreement with the reduction of CuO NPs to metallic Cu.^28^ The shoulder at a slightly higher temperature (255 °C) might originate from the reduction of larger particles or the reduction of Cu^2+^ in a stronger interaction with the silica support.^6,28^ The former explanation seems to be more probable because (i) it is consistent with XRD and STEM analyses showing the presence of both small (∼3.4 nm) and larger (∼32 nm) particles, and (ii) dry impregnation synthesis usually does not lead to strong metal–support interactions.

**Figure 4:**
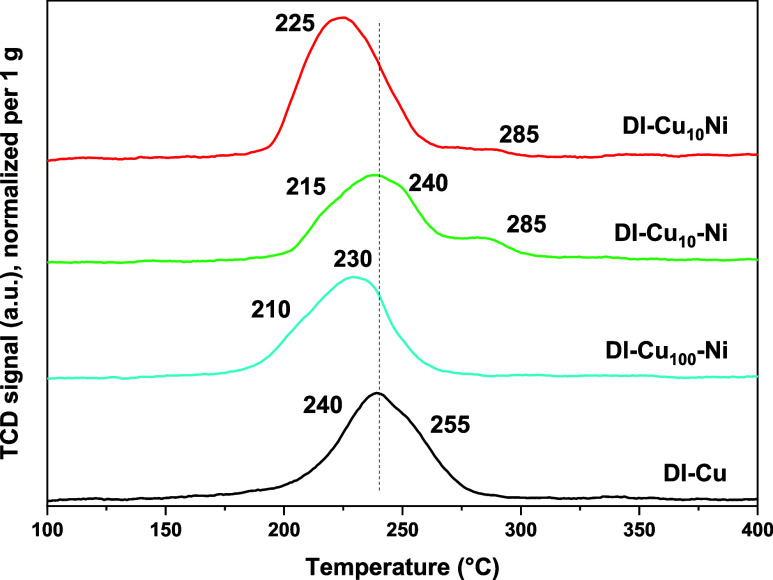
TPR profiles of Ni-doped samples. Complete TPR analyses were performed up to 1000 °C. No other reduction phenomena were observed outside the temperature range shown here.

**Figure 5:**
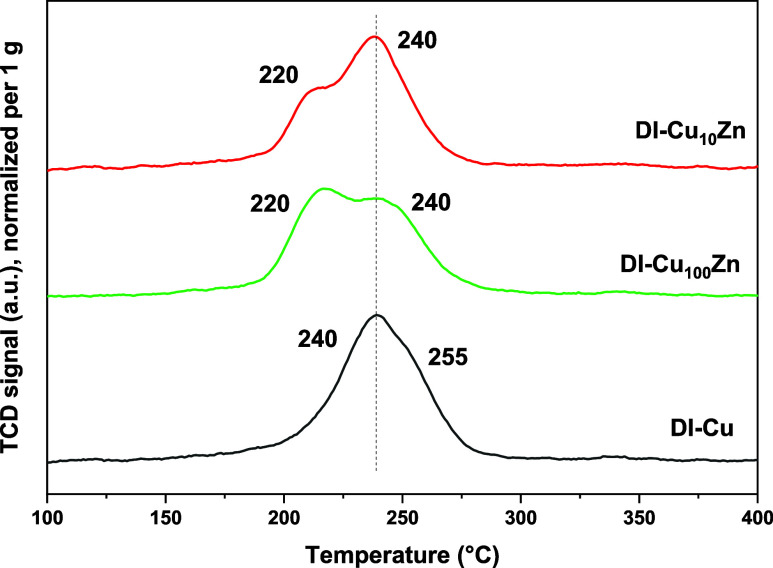
TPR profiles of Zn-doped samples. Complete TPR analyses were performed up to 1000 °C. No other reduction phenomena were observed outside the temperature range shown here.

The addition of nickel generally improved the reducibility of the materials, as the main peaks shifted to lower temperatures (210–225 °C; Figure 4). Such an effect has already been observed for low NiO loadings dissolved in CuO,^46–49^ in accordance with the limited solubility of NiO in CuO.^49^ Additional reduction phenomena have been observed at ∼285 °C for samples with a higher Ni loading (**DI-Cu**_**10**_ **Ni** and **DI-Cu**_**10**_ **-Ni**). This can be attributed to the reduction of NiO particles (not alloyed with CuO; NiO has been reported to undergo reduction at higher temperatures than CuO).^46–48^ Noteworthily, the sample prepared by one-step coimpregnation of Cu and Ni salts (**DI-Cu**_**10**_ **Ni**) exhibited a very weak, distinct high-temperature reduction peak assignable to NiO. Since no further reduction events were observed at higher temperatures, the reduction of Ni species must occur concurrently with CuO reduction. Although the low metal loadings and associated uncertainty in hydrogen consumption prevent the quantitative determination of the extent of alloying, the absence of a separate NiO-related reduction peak provides indirect evidence for the formation of more alloyed CuO–NiO particles. Conversely, the separate impregnation of Cu and Ni salts (**DI-Cu**_**10**_ **-Ni**) resulted in samples exhibiting a higher fraction of segregated NiO particles, as reflected by the presence of a higher-temperature reduction feature.

The TPR profiles of the Zn-doped catalysts (Figure 5) show that the introduction of a small amount of Zn leads to a slight shift of the Cu reduction peaks toward lower temperatures, manifested by the formation of a new low-temperature maximum (∼220 °C), indicating the enhanced reducibility of Cu species. This behavior can be attributed to the electronic and/or structural interactions between Cu and Zn, which may facilitate hydrogen activation and weaken Cu–O bonds.^50,51^ With a further increase in Zn loading (**DI-Cu**_**10**_ **Zn**), the reducibility is still enhanced in comparison to the **DI-Cu** sample. However, it slightly deteriorates in comparison to **DI-Cu**_**100**_ **Zn**, as evidenced by a shift of the most intense reduction peak back to ∼240 °C. At higher Zn contents, the formation of strongly interacting Cu–Zn–O species^52^ or partial coverage of Cu sites by ZnO can occur,^53–55^ limiting hydrogen access to Cu oxide species and thus hindering their reduction.

In conclusion, the reducibility of the Cu-based catalysts improved upon both Ni and Zn doping, as the corresponding TPR patterns showed peaks at lower temperatures in comparison to those of **DI-Cu**. Cu reducibility can play a decisive role in catalytic performance. Although Cu-based catalysts are usually reduced to metallic Cu before the catalytic tests,^46,56^ the important role of Cu^+^ in nonoxidative ethanol dehydrogenation has been shown by several authors.^23,57–59^ Also, the in situ changes in the Cu oxidation state under the ethanol stream must be taken into account.^20,56,59^ A simple experiment comparing fresh-calcined with fresh-reduced **DI-Cu**_**100**_ **Zn** samples showed that their catalytic performance at 325 °C was virtually identical (Figure S20). In addition, CuO observed in the fresh-calcined catalyst has been reduced to metallic Cu after several hours on stream (PXRD; Figure S20).

### Catalysis

Nonoxidative ethanol dehydrogenation has been studied in the temperature range of 185 to 325 °C. The stability was tested at 325 °C for 14 h directly after temperature ramping. The application of a relatively high temperature (325 °C) allows the use of a short contact time (WHSV = 4.73 h^–1^) and considers the catalytic performance also for the ethanol-to-butadiene reaction.^16,17^ Some additional stability tests were performed at 255, 290 °C, and 325 °C for the selected catalysts (see below).

The acetaldehyde selectivity increased with conversion and temperature for both the Ni- and Zn-doped samples (Table S4). It reached ∼90% for most catalysts at 220 °C, similar to the Cu-based parent catalysts (**DI-Cu**: 91%; **HSG-Cu**: 92%).^6^ Only three Ni-doped catalysts (**DI-Cu**_**10**_ **Ni**, **DI-Cu**_**100**_ **Ni**, and **DI-Cu**_**10**_ **-Ni**) exhibited a lower acetaldehyde selectivity (63–77%). Other studies have also reported a decrease of acetaldehyde selectivity in the presence of Ni particles in the catalysts.^25,30^ The carbon balance fluctuated around 100% (±5%; Table S5), confirming the high acetaldehyde selectivity of all the catalysts. The conversion of ethanol to products undetectable via FID (i.e., CO) was negligible. The reproducibility of the catalytic data is shown in Figure S21.

Zinc doping had no significant effect on the acetaldehyde selectivity during nonoxidative ethanol dehydrogenation (Table S4). Small amounts of ethylene and diethyl ether were consistently observed across the entire temperature range (185 to 325 °C) for all the catalysts. Ethyl acetate, commonly produced over Cu–ZnO catalysts (i.e., high Zn loading),^14,60^ has not been observed among the reaction products herein.

The Ni-doped samples also produced minute amounts of ethylene and diethyl ether. Additionally, traces of methane were observed above 255 °C. It has been reported that ethanol decomposes over Ni particles to CH_4_, CO, and H_2_.^25,30^ Thus, the presence of traces of methane among the reaction products agrees well with the sporadic observation of Ni-rich crystalline particles (∼20 nm, STEM, see Figure S18). The presence of separate Ni-based species has also been suggested based on the occurrence of additional reduction phenomena at ∼ 285 °C in Ni-rich samples in the TPR profiles (Figure 4).

Ni-doped catalysts prepared via two-step impregnation (**DI-Cu**_**100**_ **-Ni**, **DI-Cu**_**10**_ **-Ni**, **DI–Ni-Cu**_**10**_) showed superior activity compared to those synthesized by one-step impregnation (**DI-Cu**_**100**_ **Ni** and **DI-Cu**_**10**_ **Ni**; Figure S22). Moreover, a lower Ni content seems to be beneficial for the catalytic performance (e.g., compare **DI-Cu**_**100**_ **-Ni** with **DI-Cu**_**10**_ **-Ni**). **HSG-Cu**_**10**_ **-Ni** achieved the best catalytic conversion at 185, 220, and 255 °C. Interestingly, the parent **HSG-Cu** also outperformed **DI-Cu** and other catalysts prepared by various methods.^6^

The most active **HSG-Cu**_**10**_ **-Ni** exhibited 40–45% ethanol conversion at 220 °C, a ∼5% increase over the parent **HSG** catalyst (Figure 6, **left**).^6^ **DI-Cu**_**100**_ **-Ni** also exhibited a higher initial ethanol conversion (37 to 30% in 1 h) than the parent **DI-Cu** (30%) at 220 °C (Figure 6, **right**).^6^ The improvement in the catalytic activity of ethanol dehydrogenation upon doping Cu and other metals with Ni has already been described.^25,29,30^ The results fit well with the lower apparent activation energies and higher ethanol adsorption energies observed for the Ni-doped samples and Ni, respectively.^25,29,30^

**Figure 6:**
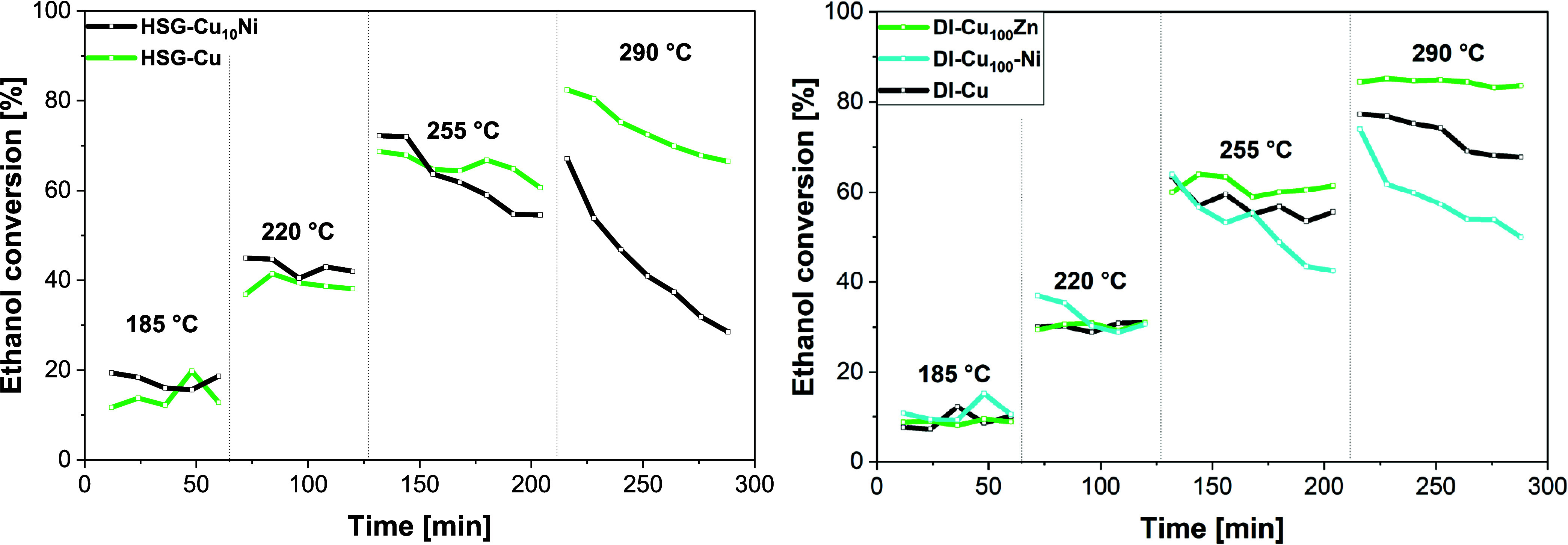
Comparison of the catalytic activities of the Ni- and Zn-doped catalysts with those of the parent DI and HSG. Each step represents one temperature (185, 220, 255, and 290 °C) with the following reaction conditions: 100 mg of catalyst, 50 mL min^–1^ N_2_, 4.73 g g^–1^ h^–1^ ethanol.

In fact, the apparent activation energy estimated for **DI-Cu100-Ni** reached ∼48 kJ mol^–1^ (Figure S23, Table S6). This value is lower than that for the **DI-Cu** sample (∼58 kJ mol^–1^) and for the catalysts based on Cu NPs on silica (65–79 kJ mol^–1^).^29^ Conversely, the observed apparent activation energy fits very well with the values obtained by Shan et al. for highly diluted CuNi alloys (45–53 kJ mol^–1^).^29^ Based on the *E*_a_comparison, it appears that Ni alloys with Cu in nickel-doped copper-based samples prepared herein, in agreement with the unlimited miscibility of Cu and Ni,^61^ and similar to catalysts reported elsewhere.^29^ Alloying of Cu with Ni has also been suggested based on the TPR results, and its intensity depended on the Ni content and synthetic procedure (Figure 4).

Nevertheless, it remains unclear why the Ni-doped samples prepared by one-step impregnation perform significantly worse than catalysts prepared by two-step impregnation, although the former exhibited more extensive alloying according to the TPR profiles. It can be hypothesized that extensive alloying leads to a lowering of the Cu active surface area, thus decreasing the catalytic activity. Unfortunately, the use of N_2_O reactive frontal chromatography (RFC N_2_O) for the Cu surface area determination is not applicable to bimetallic Cu–Ni/SiO_2_ samples when both metals are reduced within the same temperature window because N_2_O does not selectively oxidize only Cu but also oxidizes Ni.^62^

Above 220 °C, all the Ni-doped catalysts underwent rapid deactivation during the time-on-stream (TOS; Figures 6, S22). At 290 °C, the ethanol conversion over **HSG-Cu**_**10**_ **-Ni** and **DI-Cu**_**100**_ **-Ni** dropped within 1.5 h by 40% and 25%, respectively. In comparison, the parent **HSG-Cu** and **DI-Cu** lost only ∼15 and 10% of ethanol conversion, respectively, under the same conditions (290 °C).^6^ The abrupt deactivation could be connected to trace methane production (see above). It has already been observed^25,30^ that the presence of nickel particles in the catalysts leads to the partial conversion of ethanol to methane, carbon monoxide, and hydrogen. Methane pyrolysis (over nickel particles) at high temperatures is accompanied by carbon (i.e., coke) production.^63,64^ Coking can, in turn, lead to catalyst deactivation.^34^ On the contrary, highly diluted CuNi and AuNi alloys (i.e., Ni present mostly as single atoms) exhibited improved stability in ethanol dehydrogenation in comparison to Cu-based (Ni-free) catalysts.^29,30^ Based on these reports, it appears that the Ni-doped catalysts presented herein both suffer from the occasional presence of Ni-rich crystalline particles (STEM-EDS and TPR; mainly at higher temperatures) and benefit from highly dispersed/alloyed Ni clusters (STEM-EDS, TPR, and *E*_a_ of the **DI-Cu**_**100**_ **-Ni** sample; mainly at lower temperatures). The ethanol decomposition and methane pyrolysis are more pronounced at higher temperatures (≥255 °C) as Ni migration, particle formation, and pyrolysis are enhanced under these conditions.^30,63^

The Zn-doped catalysts exhibited stable catalytic performance across a 185–290 °C temperature range, in contrast to Ni-doped samples (Figure 7). The preparation method (one-step vs two-step impregnation) and Zn content (0.025 vs 0.25 wt %) had minimal impact on activity, in contrast to Ni-doped materials. **DI-Cu**_**100**_ **Zn** achieved ∼60% ethanol conversion at 255 °C, comparable to the parent **DI-Cu** catalyst (57%; Figure 6 **right**).^6^

**Figure 7:**
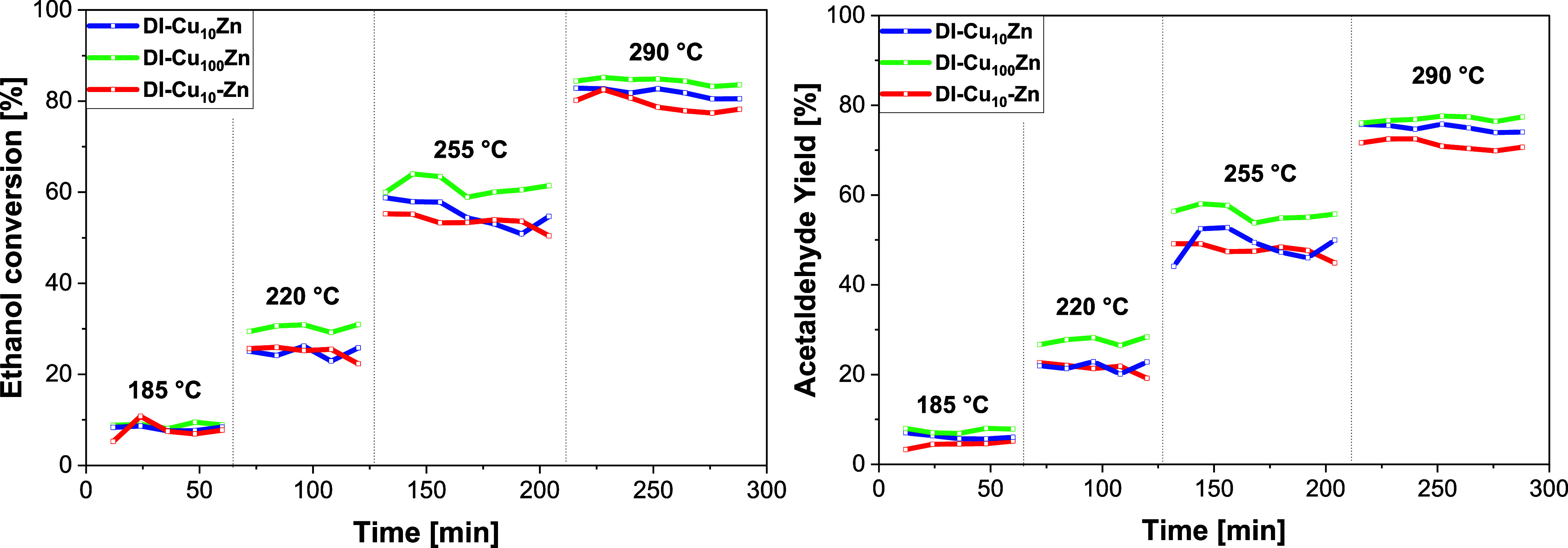
Comparison of ethanol conversion and acetaldehyde yield over Zn-doped catalysts. Each step represents one temperature (185, 220, 255, and 290 °C). Reaction conditions: 100 mg of catalyst, 50 mL/min N_2_, 4.73 g g^–1^ h^–1^ ethanol.

The similarity between the Zn-doped catalysts, particularly **DI-Cu**_**100**_ **Zn**, and the parent **DI-Cu** catalyst was further demonstrated by their apparent activation energies (∼59 and 58 kJ mol^–1^, respectively, Figure S23, Table S6). Although the TPR profiles showed an improved reducibility of Zn-doped catalysts (Figure 5), suggesting electronic and/or structural interactions between Cu and Zn,^50,51^ Zn doping did not significantly influence the ethanol conversion (Figure 6, **right**). It is surprising considering the fact that sole ZnO has recently been reported as a highly active catalyst for nonoxidative ethanol dehydrogenation.^65^

Although Zn doping did not alter the ethanol conversion or acetaldehyde selectivity, it significantly improved the catalytic stability during the temperature step tests. All the Zn-doped samples remained highly stable up to 290 °C without deactivation. **DI-Cu**_**100**_ **Zn** retained 85–83% conversion over 1.5 h at 290 °C, whereas the ethanol conversion of the **DI-Cu** parent catalyst declined from 77 to 68% under the same conditions (Figure 6). The improved stability led, in turn, to higher acetaldehyde productivity. At 290 °C, **DI-Cu**_**100**_ **Zn** exhibited the highest acetaldehyde productivity among the samples tested herein (3.63 g g^–1^ h^–1^), outperforming Ni-doped and parent catalysts (Figures 6, S22).^6^

However, a 3-day-long stability test (no temperature steps before the analysis; Figure S24) at 255 and 290 °C revealed comparable catalytic performances for both parent **DI-Cu** (Zn-free) and Zn-doped catalyst (**DI-Cu**_**100**_ **Zn**). The ethanol conversion followed a similar trend for both catalysts, exhibiting slow deactivation during the time-on-stream. Although the results were generally similar, a slight effect of zinc stabilization was observed at 255 °C. **DI-Cu**_**100**_ **Zn** showed a higher conversion (by ∼8%) compared to that of **DI-Cu** after 2.3 days.

Regeneration at 500 °C in 10% O_2_ for 1 h led to virtually complete restoration of the original catalytic activity for all tested samples (Figure S24). Therefore, it can be suggested that coke formation presents an important deactivation source. Interestingly, the regenerated Zn-doped catalyst tested at 255 °C exhibited better catalytic performance than the parent **DI-Cu** sample, showing slower deactivation. Noteworthily, parent **DI-Cu** and **DI-Cu**_**100**_ **Zn** samples tested at 290 °C exhibited a faster deactivation after the regeneration process, indicating that possible sintering of the nanoparticles and structural changes might have happened (see the Deactivation section).^34^

The data from the catalytic tests (Figures 7 and S24) suggest that zinc doping might have a positive effect on the stability of Cu catalysts in ethanol dehydrogenation, especially at lower temperatures. However, deactivation (probably caused mainly by coking) can only be slightly suppressed during long-term catalytic experiments and will be studied in more detail (see below section Deactivation).

As already noted, temperatures above 300 °C are necessary to convert ethanol to butadiene, where ethanol dehydrogenation presents the first step of the reaction pathway.^17^ Therefore, the catalytic stability of the materials prepared herein was tested at 325 °C. The Ni-doped catalysts exhibited rapid deactivation during long-term high-temperature tests at 325 °C (Figures S25 and S27). Except for **DI-Cu**_**100**_ **-Ni**, ethanol conversion dropped below 20% after 14 h, while the parent **DI-Cu** catalyst retained 44%.^6^ Nickel addition promoted coking (see the Deactivation section), accelerating deactivation,^25,34^ in good agreement with the tests performed at lower temperatures (see above).^25^ **DI-Cu**_**100**_ **-Ni** (0.085 wt % Ni, two-step impregnation) showed the highest stability among Ni-doped samples, maintaining ∼ 35% conversion. Noteworthily, **DI-Cu**_**100**_ **-Ni** is the only Ni-doped sample that did not exhibit a separate NiO reduction phenomenon during H_2_-TPR analysis (Figure 4). Still, the stability was worse compared to that of the parent **DI-Cu** (Figure 8). The highest acetaldehyde productivity at 325 °C among the Ni-doped samples was 1.09 g g^–1^ h^–1^ (**DI-Cu**_**100**_ **-Ni**), with others below 0.5 g g^–1^ h^–1^, confirming that Ni is not an ideal dopant for long-term ethanol dehydrogenation.

**Figure 8:**
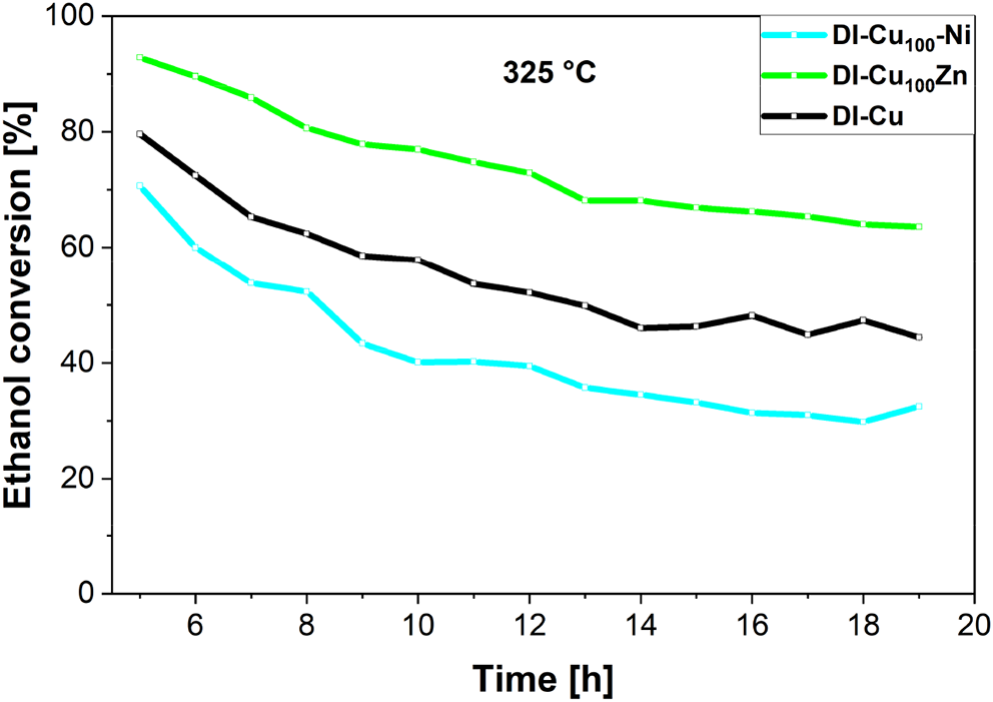
Comparison of high-temperature (325 °C) stability of **DI–Cu100-Ni** and **DI-Cu100Zn** with the parent **DI-Cu** (no Ni and Zn doping) catalyst. Ethanol conversion was analyzed under the following reaction conditions: 100 mg of catalyst, 50 mL min^–1^ N_2_, 4.73 g g h^–1^ ethanol.

In contrast, Zn doping significantly improved the stability at 325 °C (Figures S26 and S27). The preparation method influenced deactivation, where **DI-Cu**_**10**_ **-Zn** (two-step impregnation) declined from 87 to 30% conversion over 13 h, while **DI-Cu**_**10**_ **Zn** and **DI-Cu**_**100**_ **Zn** (one-step impregnation) retained higher stability after 13 h, decreasing from 92 to 51% and 93 to 64% during 14 h on TOS, respectively. Both outperformed the parent **DI-Cu** catalyst, which dropped from 79 to 44% (Figure 8).^6^ Also, the deactivation slope showed a lower progress for **DI-Cu**_**100**_ **Zn** in comparison to that of parent **DI-Cu** (−31 and −44% of initial activity, respectively).

The improved catalytic stability observed for Zn-doped catalysts during short-term catalytic runs at 255, 290, and 325 °C was also noticed during a long-term catalytic test performed at 325 °C (∼100 h) on **DI-Cu**_**100**_ **Zn** and **DI-Cu**_**100**_ **-Ni**. Regeneration at 500 °C in 10% O_2_ for 1 h led to virtually complete restoration of the original catalytic activity of the tested samples (Figure S27). Similar to the long-term tests performed at 255 and 290 °C, coke formation was suggested to be an important deactivation source. This is further supported by the observation of the very similar catalytic performances of both catalysts after regeneration. Unfortunately, deactivation could not be completely avoided and was still observed at high temperatures, even with the most stable Zn-doped catalyst (**DI-Cu**_**100**_ **Zn**). The reasons for the better catalytic stability of the Zn-doped samples compared to the parent (Cu only) and Ni-doped catalysts are discussed in the Deactivation section.

The performance of the best catalyst at 290 °C (**DI-Cu**_**100**_ **Zn**) and its acetaldehyde productivity were compared with literature results (Table 2). The published data point to the instability of copper catalysts, regardless of the preparation method, Cu loading, or particle size. **DI-Cu**_**100**_ **Zn** with 2.61 wt % Cu and 0.063 wt % Zn is one of the most active catalysts (Table 2), but deactivation occurred during the 3-day stability test. At the beginning of the test, the acetaldehyde productivity reached 3.63 g g^–1^ h^–1^ and decreased to 1.83 g g^–1^ h^–1^ after 2.2 days. A higher acetaldehyde productivity at 250 °C was observed with I–Cu7.4Si^32^, with 6.4 wt % Cu supported on silica prepared by the aerosol-assisted sol–gel technique (5.09 g g^–1^ h^–1^), but the catalytic performance was also unstable during TOS. The addition of Ni increased the catalytic activity, similar to the catalysts presented herein; however, the catalyst also underwent deactivation at 260 °C.^25^ The zinc-containing microparticle catalyst Cu30ZnAl^20^ with 19 wt % Cu exhibited only low acetaldehyde productivity (0.93 g g^–1^ h^–1^), proving the advantage of the highly active nanoparticles dispersed on the SiO_2_ support prepared by DI. The stability with TOS has not been studied in this particular case.^20^

**Table 2:** Comparison of the Catalytic Activity of the Catalysts Prepared Herein with Those of the Reported Catalysts

sample	Cu [wt %]	WHSV [h^–1^]	*T* [°C]	ethanol conversion [%]	acetaldehyde selectivity [%]	acetaldehyde productivity [g g^–1^ h^–1^]
**DI-Cu**_**100**_ **Zn** (this work)	2.61	4.73	290	84	92	3.63
**DI-Cu** ^6^ (parent catalyst)	2.42	4.73	290	77	94	3.42
Cu/SiO_2_ ^25^	25	2.37	280	67	94	1.49
Cu-Ni/SiO_2_ ^25^	25	2.37	280	77	83	1.51
Cu/SiO_2_-AE^28^	10	3.49	280	98	99	3.39
Cu/β zeolite^23^	5	1.99	300	85	82	1.39
I-Cu7.4Si^32^	6.4	9.25	250	55	100	5.09
Cu30ZnAl^20^	19	1.36	290	72	95	0.93

### Deactivation

The catalysts were characterized before and after the catalytic tests to investigate the deactivation mechanism. PXRD analyses of the spent DI-prepared samples revealed metallic Cu diffractions (98-062-7113), consistent with the reduction of CuO (98-003-1059) during pretreatment and catalysis (Figure S28). The parent **DI-Cu** catalyst was also reduced to metallic Cu,^6^ while **HSG–Cu10-Ni** remained XRD-amorphous after the catalytic tests, similar to parent HSG.^6^

The diffraction maxima, d, and lattice parameters in the spent DI-prepared samples were not shifted (Table S7), indicating no observable alloying of copper crystallites with nickel or zinc. Diffractions for separate Ni and Zn crystallites were not observed. PXRD analyses with a lower scanning rate and step size allowed us to neither confirm nor disprove Cu alloying with Ni or Zn.

Crystallite sizes, estimated using the Debye–Scherrer equation, slightly increased for all DI samples, regardless of the Ni content or preparation method. For instance, **DI-Cu**_**100**_ **-Ni** grew from 38 nm (CuO) to 41 nm (Cu^0^) during the catalysis. The Zn-doped catalysts showed a similar trend, with the crystallite size increasing from ∼25 to 40 nm. Importantly, the crystallite sizes must be considered with care, as the content of the crystalline phase is intrinsically low in the catalysts.

Although PXRD indicated the presence of Cu crystallites of ∼25–40 nm in size, STEM imaging of the spent samples (Figures 9, S29, and S30) revealed predominantly small nanoparticles (2.0–2.8 nm) with narrow size distributions (Table 3). Similar to the fresh catalysts, larger Cu particles (i.e., NP size in agreement with PXRD) were observed only rarely in the STEM images. On the one hand, electron microscopy is not a bulk method in contrast to PXRD, and allows the analysis of only a small fraction of the samples. On the other hand, extensive electron microscopy analyses have been performed to confirm the prevalent occurrence of small XRD-amorphous nanoparticles and to further support the conclusions of this work.

**Figure 9:**
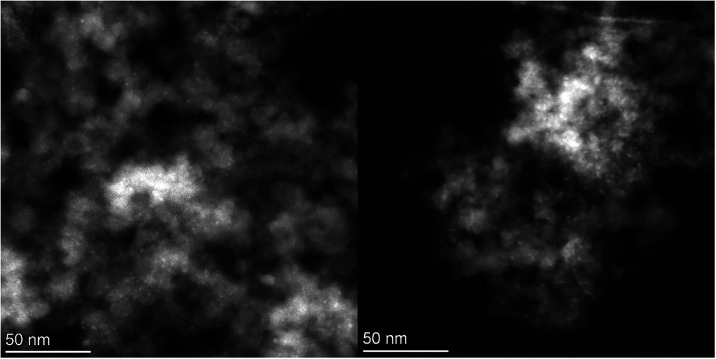
Micrographs of the spent catalysts collected by STEM of **DI-Cu**_**10**_ **-Ni** (left) and **DI-Cu**_**10**_ **Zn** (right).

**Table 3:** Nanoparticle Size Distribution and Standard Deviation by the Graphic Analysis of the Spent Catalysts (Micrograph Surveys Collected by STEM), and TG Study on the Deactivation of Ni- and Zn-Doped Catalysts by Coking

			mass change [%]				
sample	average NP diameter [nm]	standard deviation [nm]	fresh	spent	coking by TG [%]	onset temperature [°C]	C/Cu ratio^a^ (−)
**HSG-Cu**_**10**_ **-Ni**	2.3	0.8	1.31	4.36	**+3.05**	423	6.74
**DI-Cu**_**10**_ **Ni**	1.9	0.4	0.37	0.72	**+0.35**	440	5.60
**DI-Cu**_**100**_ **Ni**	2.0	0.5	0.50	2.53	**+2.03**	423	8.08
**DI-Cu-Ni**_**10**_	1.9	0.6	0.93	2.69	**+1.76**	423	8.27
**DI-Cu-Ni**_**100**_	2.1	0.4	0.61	2.31	**+1.70**	427	9.04
**DI-Ni**_**100**_ **-Cu**	2.4	0.6	0.51	2.61	**+2.10**	429	8.73
**DI-Cu**_**10**_ **Zn**	2.1	0.5	0.53	1.47	**+0. 94**	380	6.40
**DI-Cu**_**100**_ **Zn**	2.8	0.5	0.50	2.06	**+1.56**	392	6.18
**DI-Cu**_**10**_ **-Zn**	2.1	0.4	0.53	1.35	**+0.82**	375	5.25

a Based on the C and Cu wt % from the XPS analyses.

The particle sizes remained nearly unchanged compared to the fresh catalysts, independent of the Ni or Zn content (STEM). For example, **DI-Cu**_**100**_ **Zn** exhibited particle sizes of 2.8 nm (σ = 0.5 nm) after the reaction, similar to 2.6 nm (σ = 0.7 nm) in the fresh state. The parent **DI-Cu** catalyst also retained comparable particle sizes (2.5 nm and σ = 0.5 nm), indicating minimal particle growth during the time-on-stream. The addition of Ni and Zn does not seem to affect the sintering and growth of the nanoparticles. No significant changes were observed in the STEM-EDS elemental mapping of the spent catalysts, and neither separation nor sintering of nickel and zinc was observed (Figures S31 and S32). All three metals, copper, nickel, and zinc, were homogeneously dispersed over the silica support. Thus, particle sintering, growth, and metal separation do not appear to be significant sources of deactivation. This can be further supported by the regeneration experiments (Figure S27), which lead to (i) complete restoration of the catalytic activity and (ii) similar deactivation patterns after regeneration.

XPS analysis of the spent catalysts corroborated the STEM-EDS results. A decrease of the Cu/Si ratio of a catalyst after a catalytic experiment would indicate the occurrence of sintering and related phenomena.^33^ However, a comparison of the Cu/Si ratios in the fresh and spent catalysts showed no decrease in any of the materials prepared by dry impregnation (both nickel and zinc doped; Table S8). The steadiness of the Cu/Si ratios points to the stability of the Cu NPs in the catalysts against sintering during the catalytic experiments.

In contrast, the regeneration tests performed during the catalytic experiments (see the section Catalysis) suggest that the formation of carbonaceous fragments on the catalyst surface was not prevented in both cases (Ni and Zn doping). It can also play an important role in pore blockage, decrease SA_BET_ and *V*_total_, and consequently, deactivation.^68^ Furthermore, the deposited coke can be analyzed by XPS, TGA, and Raman spectroscopy.

The nitrogen adsorption measurement data obtained for the spent Ni-doped samples (Table 4) revealed significant porosity changes during catalysis. The most significant surface area loss (−41%) was observed for **HSG-Cu**_**10**_ **-Ni**, correlating with its high surface area, small pores, as well as its rapid deactivation. In contrast, the catalysts supported on Aerosil 300 showed lower SA_BET_ losses (1–20%), aligning with the higher catalytic stability in ethanol dehydrogenation in comparison to **HSG-Cu**_**10**_ **-Ni**. The two-step impregnation samples (**DI-Cu**_**10**_ **-Ni**, **DI-Cu**_**100**_ **-Ni**) exhibited minimal SA_BET_ reduction (−1 and −3%, respectively), aligning with their higher catalytic stability. The most stable Ni-doped catalyst, **DI-Cu**_**100**_ **-Ni**, also showed only a slight change in the pore volume (−9%).

**Table 4:** N_2_ Porosimetry Study on the Porosity Changes between the Fresh and Spent Ni- and Zn-Doped Catalysts

	surface area (m^2^ g^–1^)			pore volume (cm^3^ g^–1^)			pore size distribution (nm)		
preparation method	fresh	spent	surface area change (%)	fresh	spent	pore volume change (%)	fresh	spent	pore size change (%)
**HSG-Cu**_**10**_ **-Ni**	487	288	–41	0.61	0.36	–41	4.97	4.97	0
**DI-Cu**_**10**_ **Ni**	279	224	–20	1.27	0.49	–61	18.3	8.68	–53
**DI-Cu**_**100**_ **Ni**	261	249	–5	1.06	0.47	–56	16.2	7.55	–54
**DI-Cu**_**10**_ **-Ni**	219	213	–3	0.43	0.39	–9	7.90	7.21	–9
**DI-Cu**_**100**_ **-Ni**	240	238	–1	0.53	0.49	–8	8.84	8.20	–7
**DI-Ni-Cu**_**100**_	278	244	–12	1.20	0.52	–57	17.3	8.57	–50
**DI-Cu**_**10**_ **Zn**	293	287	–2	1.41	0.84	–40	19.3	11.7	–39
**DI-Cu**_**100**_ **Zn**	326	276	–15	1.85	1.65	–11	22.8	24.0	+5
**DI-Cu**_**10**_ **-Zn**	265	157	–41	0.63	0.37	–41	9.51	9.32	–2

The Zn-doped catalysts followed similar trends. The two-step impregnation sample (**DI-Cu**_**10**_ **-Zn**) showed the highest SA_BET_ and *V*_total_ losses (−41 and −41%, respectively), correlating with its lower stability. In contrast, the one-step impregnation samples (**DI-Cu**_**10**_ **Zn**, **DI-Cu**_**100**_ **Zn**) demonstrated better stability, with a significantly lower decrease of the specific surface area (Table 4).

The C/Cu ratios obtained from the XPS analyses could be used as another indicator of coking behavior (Table 3). The catalysts can be safely divided into two groups: (i) materials with a lower C/Cu ratio (5.25–6.40), and (ii) catalysts with a higher C/Cu ratio (6.74–9.04). All Zn-doped samples exhibited a lower C/Cu ratio. On the contrary, the Ni-doped samples showed a higher C/Cu ratio. The only exception was **DI-Cu**_**10**_ **Ni**. Its poor catalytic activity resulted in a lower amount of deposited coke.

The extent of coking was further estimated by TG analysis of the spent catalysts (Table 3). The smallest deactivation and the lowest amount of carbon formed (0.38%) were observed in the case of the sample exhibiting poor catalytic activity (**DI-Cu**_**10**_ **Ni**; in agreement with the XPS analyses). On the contrary, the highly active sample **HSG-Cu**_**10**_ **-Ni** produced the most significant amount of coke (TG: 3.05%). This correlated with the rapidly decreasing catalytic activity (Figures 6 and S25). Cu-based parent catalysts exhibited similar results: coking was observed in **HSG-Cu** to a greater extent in comparison to **DI-Cu**.^6^

The Ni-doped samples prepared by DI exhibited a higher catalytic activity at lower temperatures than the parent sample and produced relatively similar amounts of coke in the range of 1.70—2.10% (except for poorly active **DI-Cu**_**10**_ **Ni**). On the other hand, the Zn-doped samples exhibited improved stability with TOS in comparison to the parent **DI-Cu**. Accordingly, the deposited amount of coke reached lower values (0.82—1.56%) than that of the Ni-doped samples (Table 3). The highest amount of coke among the Zn-doped samples was observed for the most active sample (**DI-Cu**_**100**_ **Zn**). These results are in excellent agreement with the C/Cu ratios derived from XPS analyses.

Interestingly, the onset temperatures of coke combustion were systematically different for the Ni- and Zn-doped samples according to the TGA analysis (Table 3, Figure 10). The Zn-containing samples exhibited lower onset temperatures (375–392 °C) in comparison to the Ni-doped samples (423–440 °C). These results suggest that not only the amount but also the nature of the coke deposited on the Zn- and Ni-doped samples differed.

**Figure 10:**
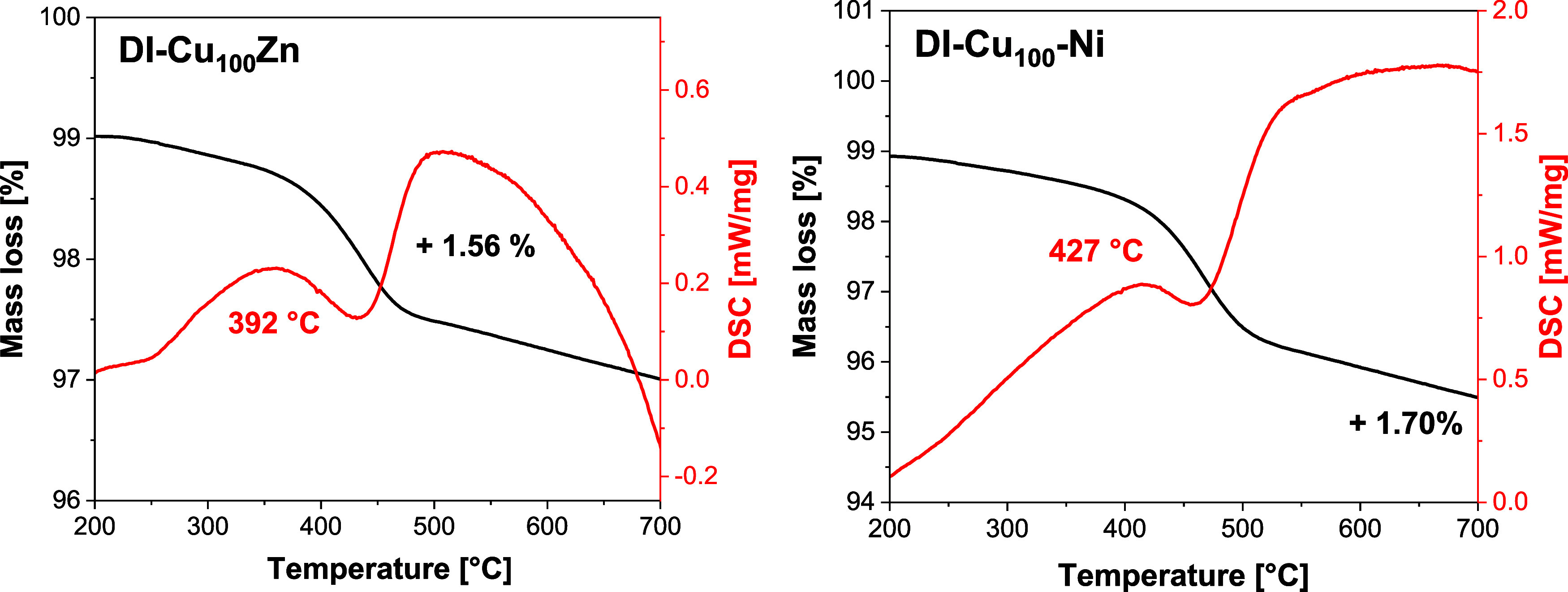
TGA analyses of the DI-Cu_100_Zn and DI-Cu_100_-Ni samples showing mass losses and onset temperatures of coke combustion.

Raman spectra of the spent samples were collected to further describe the coke deposited on the catalysts (Figure 11). The so-called G- and D-bands were observed in all spectra at ∼1590 and 1360 cm^–1^, respectively. Although the former band originates from the vibrations of an ideal graphitic lattice, the latter is related to structural defects occurring in graphitic carbon.^69,70^ Thus, the spectra confirmed the formation of graphitic carbon in the samples during catalysis. The *I*_D_/*I*_G_ ratio fluctuates between 0.9 and 1.0 for all samples, indicating the formation of highly defective graphitic carbon during catalysis. Nevertheless, the overall intensity of the bands suggests that the amount of deposited coke is higher in the Ni-doped catalysts in comparison to Zn-doped samples, in agreement with the XPS and TG analyses.

**Figure 11:**
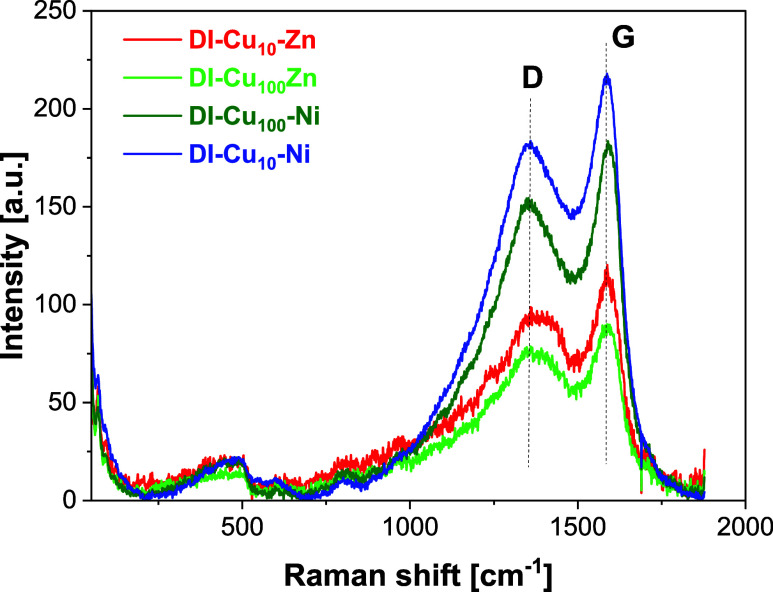
Raman spectra of the spent catalysts **DI-Cu**_**10**_ **-Zn**, **DI-Cu**_**100**_ **Zn**, **DI-Cu**_**100**_ **-Ni**, and **DI-Cu**_**10**_ **-Ni**.

It has already been discussed that ZnO was reported to work mainly as a structural promoter; it stabilizes Cu NPs against sintering, thereby improving the stability of the Cu-based catalysts.^31^ Herein, sintering does not seem to be the most significant cause of deactivation (STEM, see above). Instead, coking appears to be the major source of deactivation, similar to the study by Pampararo et al.^33^ Based on the characterization of the spent catalysts, the better stability of the Zn-doped samples might be related to a lower extent of coking (XPS, TGA, and Raman spectroscopy; see above). Similarly, the addition of ZnO has been shown to improve the stability of the CoO catalysts in propane dehydrogenation as it suppressed coking.^70^ To the best of our knowledge, here, we report for the first time that Zn doping modifies the coking behavior of Cu-based ethanol dehydrogenation catalysts.

The better coking resistance of the Zn-doped samples might originate either from different adsorption properties toward the reaction intermediates prone to form coke or from the modification of Cu properties upon ZnO doping (i.e., the decrease of Cu site activity for carbon deposit formation). The present data in hand do not allow us to unambiguously confirm any of these suggestions. However, it is worth noting that the most stable catalyst, **DI-Cu_100_Zn**, exhibited the best reducibility among the Zn-doped samples and the most different TPR pattern from the parent **DI-Cu** sample. Conversely, the sample **DI-Cu_10_Zn** exhibited lower stability than **DI-Cu_100_Zn** and a TPR pattern more similar to that of the **DI-Cu** sample.

## Conclusion

This work demonstrates the effects of zinc and nickel doping on the activity and stability of Cu-based catalysts deposited on the porous silica support using dry impregnation (DI) and hydrolytic sol–gel (HSG) synthesis methods. Nonoxidative ethanol dehydrogenation was studied as the intended catalytic reaction. STEM analyses revealed mainly small particles on the silica support (from 1.9 to 2.9 nm) with only rare formation of larger Cu crystallites. PXRD analysis confirmed that these inhomogeneities originated from the dry impregnation method (DI) with occasional metal agglomeration (12–28 nm crystallite sizes).

Nonoxidative dehydrogenation was carried out in the temperature range of 185 to 325 °C, with the selectivity to acetaldehyde fluctuating around 90%. This study shows that highly diluted Ni slightly promotes the reactivity of copper deposited on silica at lower temperatures (e.g., 185 and 220 °C), but coking caused by Ni leads to the rapid deactivation of the catalysts at temperatures higher than 250 °C. Mixing Cu with Zn shows promising results in the range of 185 to 290 °C, where highly diluted zinc (**DI-Cu**_**100**_ **Zn**, 0.063 wt %) was able to stabilize the catalysts with promising productivity (290 °C: 3.63 g g^–1^ h^–1^). Zinc doping did not completely stabilize high-temperature catalysis at 325 °C, but significantly mitigated the deactivation rate (−31 and – 44% loss of the initial activity for **DI-Cu**_**100**_ **Zn** and zinc-free parent DI-Cu, respectively).

On one hand, extensive STEM analyses displayed very similar particle sizes and particle size distributions for all fresh and spent Ni- and Zn-doped samples. Thus, particle sintering does not appear to be a significant cause of catalyst instability during the time-on-stream. On the other hand, coking has been identified as the most important source of catalyst deactivation by XPS, TG, N_2_ adsorption–desorption experiments, and regeneration catalytic experiments. More stable Zn-doped samples formed less coke than Ni-doped catalysts, according to TG analysis and Raman spectroscopy. Although ZnO has usually been considered as a structural promoter in recent reports (i.e., it provides improved stability against sintering), here we show that it also suppresses coking and influences the catalytic behavior to a significant extent.

## Supplementary Material



## Notes

Description of syntheses (dry impregnation and hydrolytic sol–gel); apparent activation energy estimation; XPS spectra, nitrogen porosimetry data, isotherms and pore size distributions; XRD diffractograms and the corresponding data; lattice parameters, STEM micrographs, STEM-EDS elemental maps, catalytic data; conversion, selectivity, and carbon balance (PDF)
