# Supplementary material for: Ni- and Zn-Doping Effects on Cu/SiO2 Catalysts in Nonoxidative Ethanol Dehydrogenation
Source: Ind Eng Chem Res. 2026 Feb 24;65(9):4716–30. doi: 10.1021/acs.iecr.5c04241 (PMC12983309; doi:10.1021/acs.iecr.5c04241)
Supplement: Supplementary file 1 [file ie5c04241_si_001.pdf]

Ni and Zn doping effect of Cu/SiO_2 catalysts in nonoxidative ethanol dehydrogenation

Supplementary information

Tomas Pokorny,[1] Petr Machac,[1] Zdenek Moravec,[1] Lucie Simonikova,[1] Lucie Leonova,[1] Zuzana Hlavenkova,[2] David Skoda,[3] Katerina Pacultova,[4] Katerina Karaskova,[4] Ales Styskalik[1]*

[1]Department of Chemistry, Masaryk University, Kotlarska 2, CZ-61137 Brno, Czech Republic

[2]CEITEC, Masaryk University, Kamenice 5, CZ-62500 Brno, Czech Republic

[3]Centre of Polymer Systems, Tomas Bata University in Zlin, Tr. T. Bati 5678, CZ-76001 Zlin, Czech Republic

[4]Institute of Environmental Technology, CEET, VSB-TUO, 17. listopadu 2172/15, CZ-70800 Ostrava, Czech Republic

*Corresponding author (styskalik@chemi.muni.cz)

Preparation of Ni-doped Cu-based catalysts

Hydrolytic sol-gel combined with dry impregnation of Ni precursor (**HSG-Cu_{10}-Ni**): SiO_2 containing copper was prepared by hydrolytic sol-gel from $Si(OC_2H_5)_4$.[1] $Cu(NO_3)_2 \cdot 5/2H_2O$ was used as a copper precursor. All chemical species were mixed in the beaker (molar ratio TEOS:EtOH: H_2O 1:3.85:10.2), and the Cu precursor was calculated to form 2.5 wt% of Cu in the resulting SiO_2 and was added into the reaction mixture reaction.

Reaction pre-gelled for 12 hours on the air. The excess of concentrated NH_3 was quickly added into the reaction mixture after 12 hrs to achieve the final gelation. The resulting blue gel was dried in an oven at 70 °C. The xerogel was then calcined in the tube furnace. The calcined product was properly ground, and 0.660 g was used for further preparation. The dry impregnation method was used to introduce Ni (8.9 mg $NiCl_2·6H_2O$, 0.038 mmol in 6.6 cm^3 H_2O) to prepare a nominal 0.25 wt% of Ni loading (sample denoted **HSG-Cu10-Ni** as the nominal weight ratio Cu:Ni equals 10). Nickel precursor was dissolved in water and then mixed with silica containing Cu prepared by hydrolytic sol-gel method. The sample was dried in an oven at 70 °C with occasional stirring and calcined (5 h, 500 °C, 10 °C min^{-1}).

One-step dry impregnation of Cu and Ni precursors (**DI-Cu_{10}Ni** and **DI-Cu_{100}Ni**): The samples were prepared by dry impregnation (both Cu and Ni in one step). The samples contained 0.25 wt% and 0.025 wt% nominal Ni loading, respectively. Nominal Cu loading was 2.5 wt%, thus the Cu:Ni weight ratio equals 10 and 100, respectively, and samples are denoted as **DI-Cu_{10}Ni** and **DI-Cu_{100}Ni**. $Cu(NO_3)_2·5/2H_2O$ and $NiCl_2·6H_2O$ were dissolved in 30 ml of distilled water and then thoroughly mixed with silica support. In such a way a paste was prepared. Samples in the form of paste were dried at 70 °C with occasional stirring and calcined (5 h, 500 °C, 10 °C min^{-1}). (**DI-Cu_{10}Ni**: 3.00 g of Aerosil 300 ; 273.0 mg, 1.174 mmol of $Cu(NO_3)_2·5/2H_2O$; 30.4 mg, 0.138 mmol of $NiCl_2·6H_2O$. **DI-Cu_{100}Ni**: 3.00 g of Aerosil 300, 273.7 mg, 1.177 mmol of $Cu(NO_3)_2·5/2H_2O$; 3.1 mg, 0.013 mmol of $NiCl_2·6H_2O$.)

Two-step dry impregnation (**DI-Cu_{10}-Ni**, **DI-Cu_{100}-Ni**, **DI-Ni-Cu_{100}**; two-step impregnation indicated by the additional dash between Cu and Ni): The deposition of each metal was carried out separately. For samples **DI-Cu_{10}-Ni** and **DI-Cu_{100}-Ni**, Aerosil silica support was mixed with the solution of $Cu(NO_3)_2·5/2H_2O$ in 50 ml distilled water

(nominal Cu loading 2.5 wt%, 5.00 g of Aerosil 300, 457,5 mg, 1.967 mmol of $Cu(NO_3)_2 \cdot 5/2H_2O$). Samples in the form of paste were dried at 70 °C with occasional stirring and calcined (5 h at 500 °C, 10 °C min^{-1}). The same operations were performed with a solution of $NiCl_2 \cdot 6H_2O$, and the catalysts were calcined again (**DI-Cu$_{10}$-Ni**: 0.5 g of Cu-impregnated Aerosil 300; 5 ml of distilled water; 5.1 mg, 0.022 mmol of $NiCl_2 \cdot 6H_2O$. **DI-Cu$_{100}$-Ni**: 0.5 g of Cu-impregnated Aerosil 300; 5 ml of distilled water; 0.5 mg, 0.002 mmol of $NiCl_2 \cdot 6H_2O$). Nominal wt% of Ni were 0.25 wt% for **DI-Cu$_{10}$-Ni** and 0.025 wt% for **DI-Cu$_{100}$-Ni**. The **DI-Ni-Cu$_{100}$** sample was prepared in the opposite order, Ni was deposited first and then Cu, with 0.025 wt% Ni nominal concentration. First impregnation: 1 g of Aerosil 300; 10 ml of distilled water; 1.0 mg, 0.004 mmol of $NiCl_2 \cdot 6H_2O$; Second impregnation: 0.860 mg of Ni impregnated Aerosil 300; 8.6 ml of distilled water; 79.6 mg, 0.342 mmol of $Cu(NO_3)_2 \cdot 5/2H_2O$).

Preparation of Zn-doped Cu-based catalysts

One-step dry impregnation of Cu and Zn precursors (**DI-Cu$_{10}$Zn** and **DI-Cu$_{100}$Zn**): The samples were prepared by dry impregnation (both Cu and Zn in one step). The samples contained 0.25 wt% and 0.025 wt% nominal Zn loading, respectively (the nominal Cu loading was 2.5 wt%, thus the Cu:Zn weight ratio equals 10 and 100, respectively, and the samples were denoted as **DI-Cu$_{10}$Zn** and **DI-Cu$_{100}$Zn**). The preparation procedure was the same as for samples **DI-Cu$_{10}$Ni** and **DI-Cu$_{100}$Ni** (see above). 1.000 g of Aerosil 300; 10 ml of distilled water; 91.5 mg, 0.394 mmol of $Cu(NO_3)_2 \cdot 5/2H_2O$; 10.3 mg, 0.035 mmol of $Zn(NO_3)_2 \cdot 6H_2O$ was used for the preparation of **DI-Cu$_{10}$Zn**. 1.362 g of Aerosil 300; 14 ml of distilled water; 124.7 mg, 0.536 mmol of $Cu(NO_3)_2 \cdot 5/2H_2O$; 1.4 mg, 0.005 mmol of $Zn(NO_3)_2 \cdot 6H_2O$ was used for the synthesis of **DI-Cu$_{100}$Zn**.

Two-step dry impregnation (**DI-Cu$_{10}$-Zn**): Sample preparation followed the procedure used for **DI-Cu$_{10}$-Ni** (see above). 0.777 g of Aerosil 300, 8 ml of distilled water, 71.8 mg, 0.309 mmol of $Cu(NO_3)_2 \cdot 5/2H_2O$ was used for the first step of preparation. 0.722 g of Cu impregnated Aerosil 300; 8 ml of distilled water and 7.4 mg, 0.025 mmol of $Zn(NO_3)_2 \cdot 6H_2O$ was applied for the second step.

Apparent activation energy estimation

Total amount of active surface atoms (Cu_{surf}) was calculated by **eq. 1**. It depends on ratio of surface atoms to total amount of atoms in particles (D_{Cu}) and on total molar amount of Cu in the catalysts (n_{Cu}). The ratio D_{Cu} can be further determined as dependence of Cu molar volume ($V_m = 7.09 \cdot 10^{21}$ nm^3) on Cu molar area ($A_m = 4.10 \cdot 10^{22}$ nm^3) and average particles diameter (d).[2] The average particle diameters by graphic analysis of STEM micrographs were used as d in the calculations (Table 3 in the main text of the manuscript).

Eq. 1

$$Cu_{sur} = D_{Cu} \cdot n_{Cu} = \frac{6 \cdot V_m}{A_m \cdot d}$$

Turn-over-frequency (TOF) (acetaldehyde produced per time per Cu surface atom) was calculated based on **eq. 2**, representing reaction rate of acetaldehyde per hour in moles (r) divided by moles of Cu surfaces atom in catalyst (Cu_{surf}).

Eq. 2

$$TOF = \frac{r}{Cu_{surf}}$$

The apparent activation energy (E_a) was determined from the temperature dependence of the acetaldehyde formation rate according to the Arrhenius equation (**eq. 3**) and taking the natural logarithm (**eq 4.**).

Eq. 3

$$k = Ae^{\frac{-E_a}{R \cdot T}}$$

Eq. 4

$$\ln(k) = -E_a \cdot \frac{1}{R \cdot T} + \ln(a)$$

where k represents the reaction rate constant obtained as the measured rate of acetaldehyde formation (k = TOF), R is the universal gas constant (8.314 $J \cdot mol^{-1} \cdot K^{-1}$), and T is the reaction temperature in Kelvin.

Experimental reaction rates were measured at three different temperatures (185, 220, and 255 °C) under differential conditions, and the logarithm of the rate was plotted against the reciprocal temperature and gas constant ($1/T \cdot R$). To simplify the values on the x-axis and to express the slope directly in $kJ \cdot mol^{-1}$, the value was multiplied by 1000, resulting in the dependence of ln(r) on $1000/T \cdot R$ [$mol \cdot kJ^{-1}$].

For the Arrhenius plots, the particle sizes of the spent catalysts were used to better reflect the actual active surface during reaction. For stable catalysts, the average productivity was used to calculate the rate, while during unstable conditions, only the initial productivity was considered due to progressive deactivation. The apparent activation energy (E_a) was then derived from the slope of the linear fit.

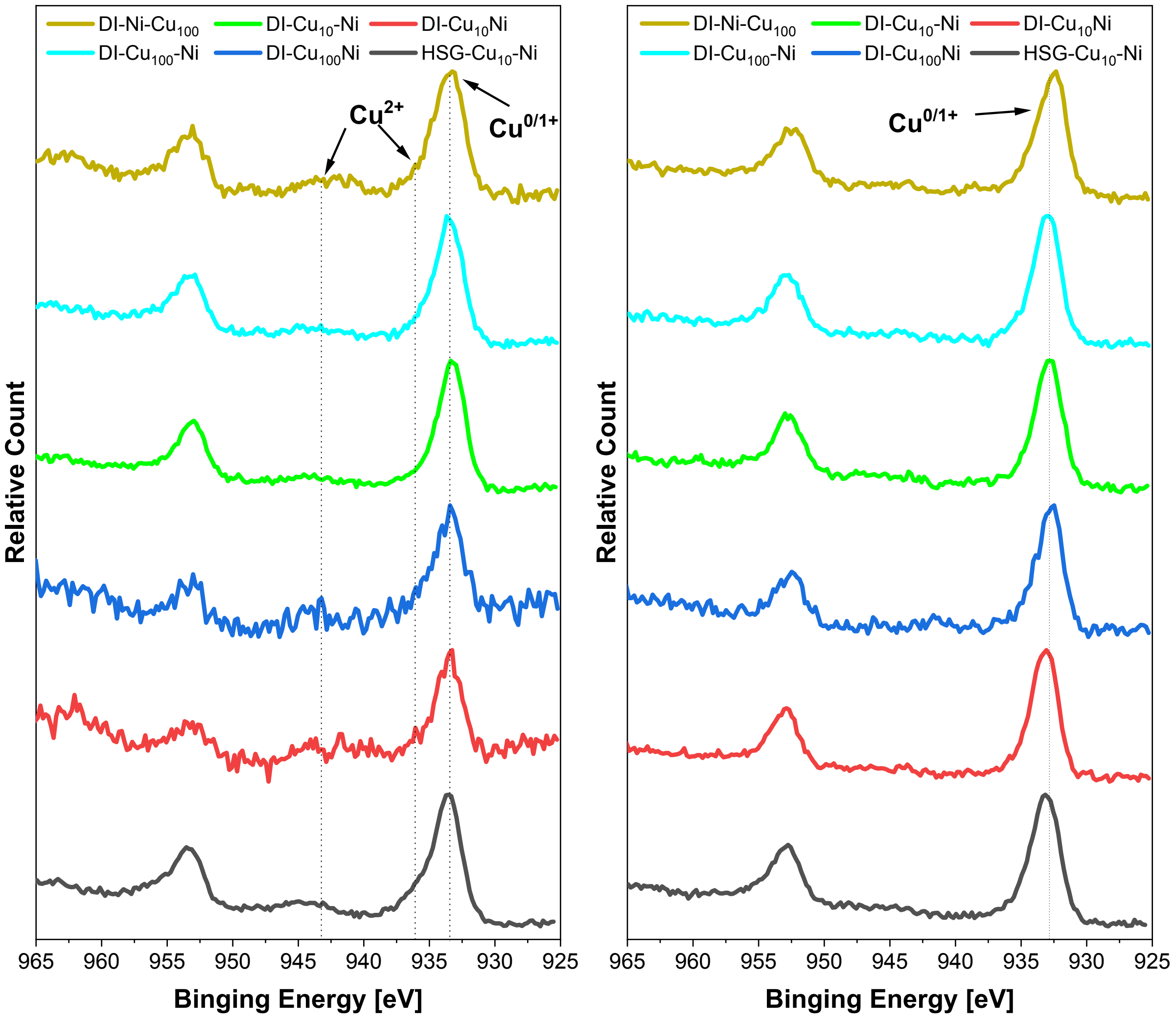

Figure S1. *Cu 2p XPS spectra of Ni-doped catalysts (left: fresh-calcined, right: spent)*

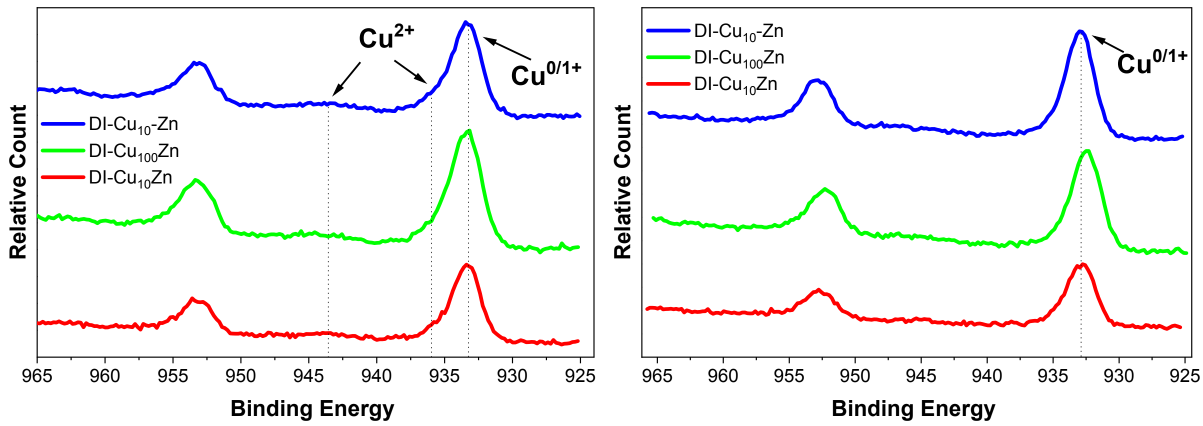

__Figure S2.__ Cu 2p XPS spectra of Zn-doped catalysts (left: fresh-calcined, right: spent)

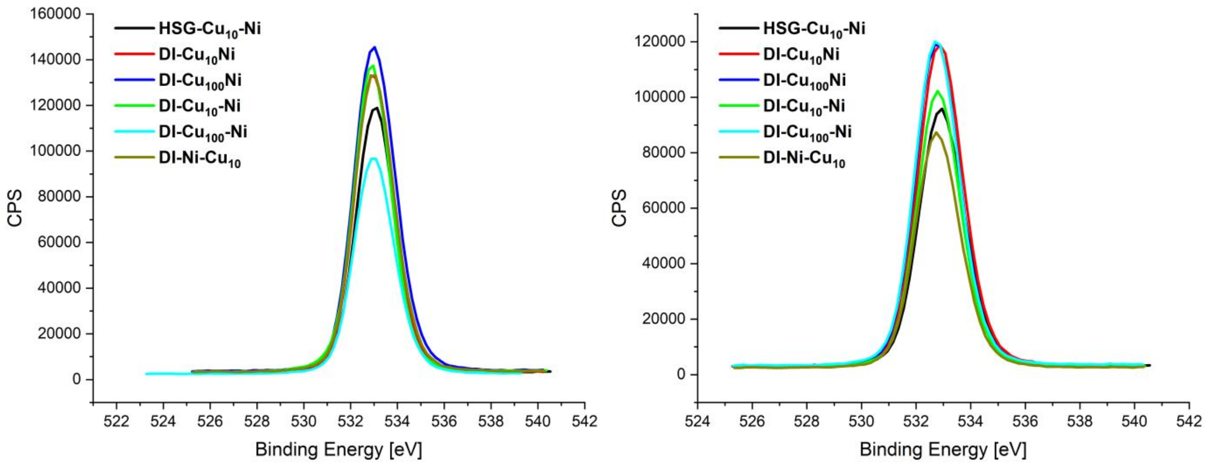

__Figure S3.__ XPS spectra of O 1s of Ni-doped catalysts (left: fresh-calcined, right: spent)

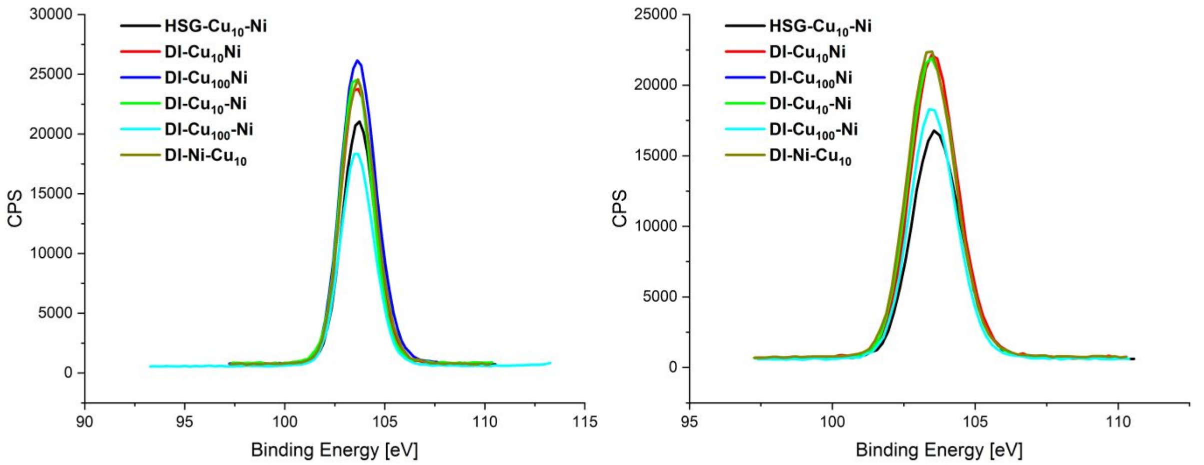

__Figure S4.__ XPS spectra of Si 1s of Ni-doped catalysts (left: fresh-calcined, right: spent)

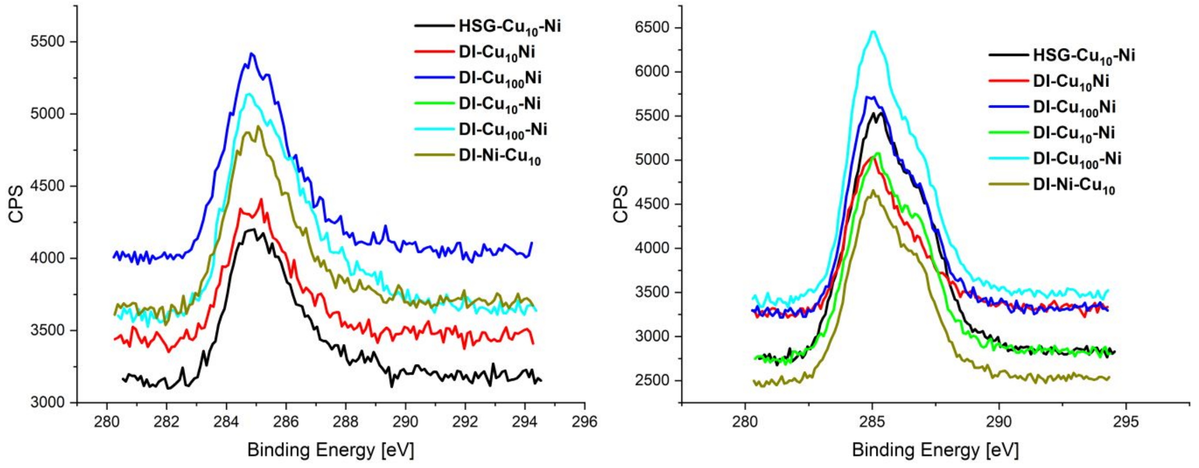

__Figure S5.__ XPS spectra of C 1s of Ni-doped catalysts (left: fresh-calcined, right: spent)

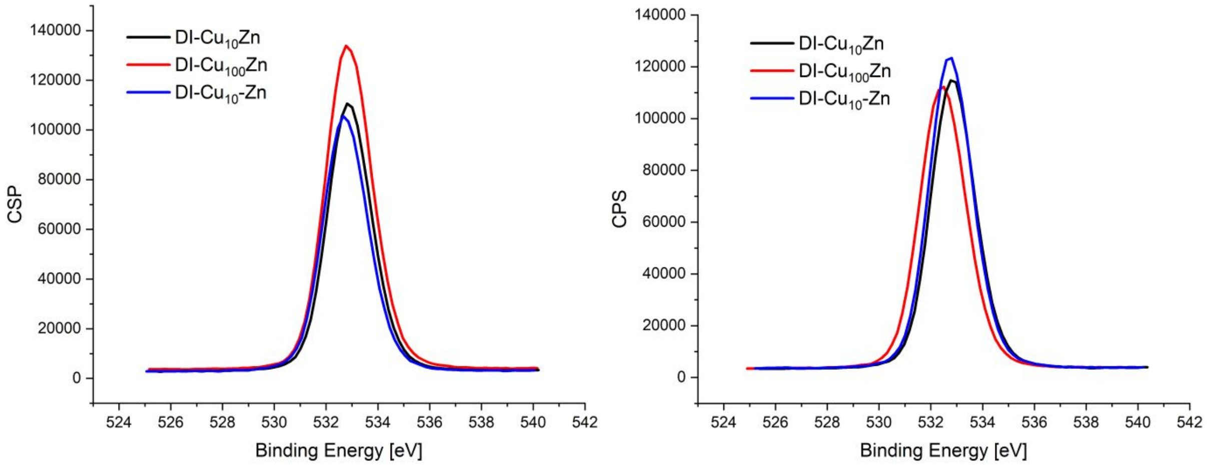

__Figure S6.__ XPS spectra of O 1s of Zn-doped catalysts (left: fresh-calcined, right: spent)

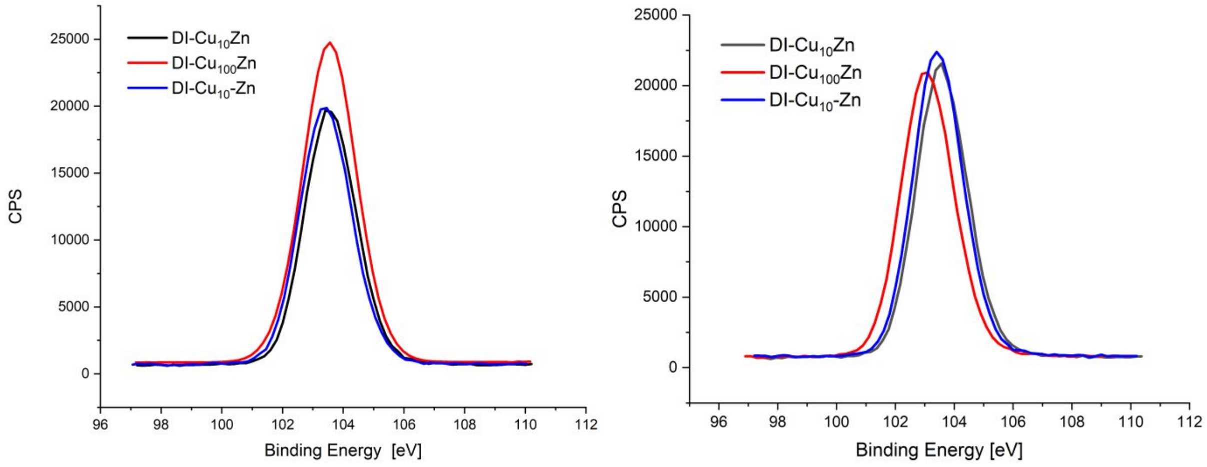

__Figure S7.__ XPS spectra of Si 2 p of Zn-doped catalysts (left: fresh-calcined, right: spent)

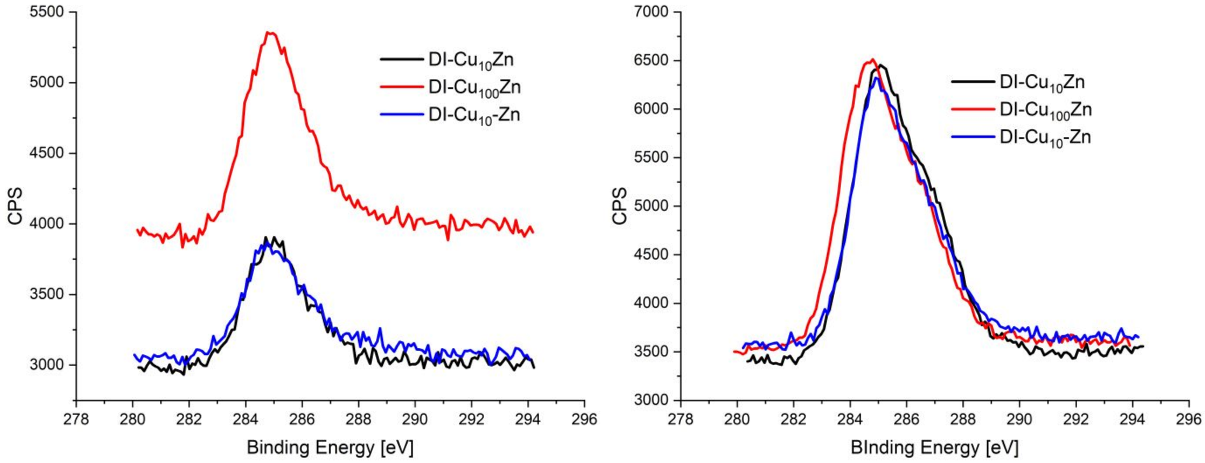

__Figure S8.__ XPS spectra of C 1s of Zn-doped catalysts (left: fresh-calcined, right: spent)

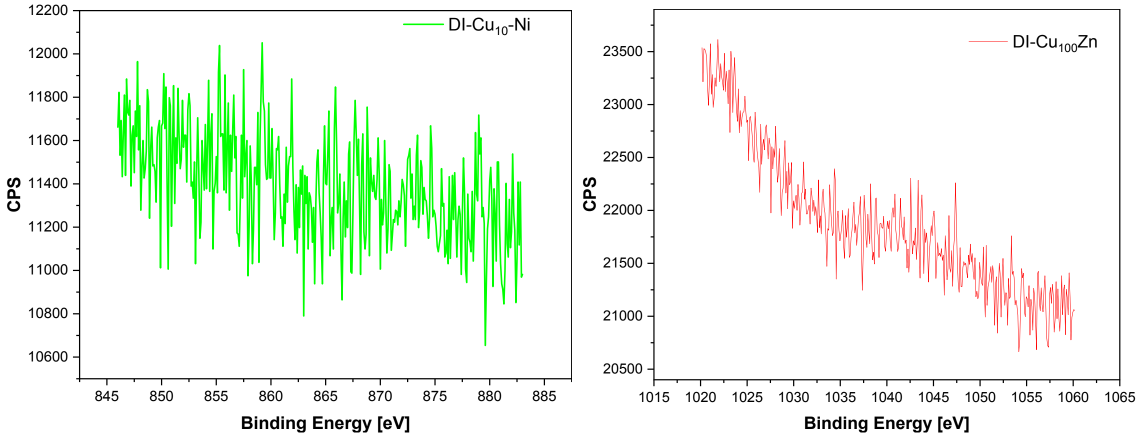

Figure S9. XPS spectra of Ni 2p (left) and Zn 2 p (right).

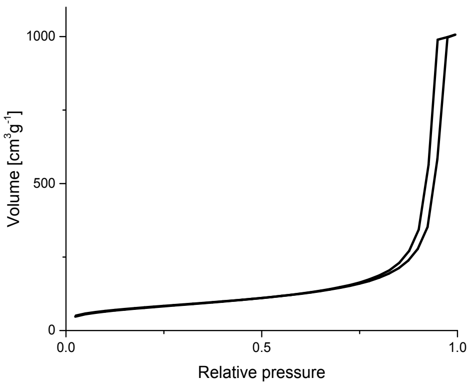

__Figure S10.__ Nitrogen adsorption and desorption isotherm of silica support Aerosil 300 used for the deposition of copper. Surface area by BET reached 284 $m^2\ g^{-1}$, pore volume 1.55 $cm^3\ g^{-1}$and average pore size 10.8 nm.

Table S1. Characterization of surface for all catalysts by N_2 adsorption porosimetry.

Preparation method	Surface area ($m^2\,g^{-1}$)	Pore volume ($cm^3\,g^{-1}$)	Average pore size[a] (nm)
**HSG-Cu_{10}-Ni**	487	0.61	4.97
**DI-Cu_{10}Ni**	279	1.27	18.3
**DI-Cu_{100}Ni**	261	1.06	16.2
**DI-Cu_{10}-Ni**	219	0.43	7.90
**DI-Cu_{100}-Ni**	240	0.53	8.84
**DI-Ni-Cu_{100}**	278	1.20	17.3
**DI-Cu_{10}Zn**	293	1.41	19.3
**DI-Cu_{100}Zn**	326	1.85	22.8
**DI-Cu_{10}-Zn**	265	0.63	9.51

[a] $4V_{total}/SA_{BET}$

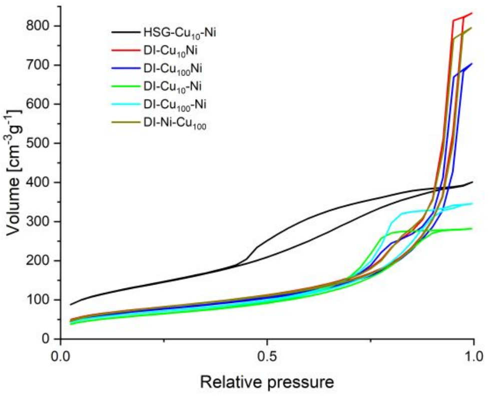

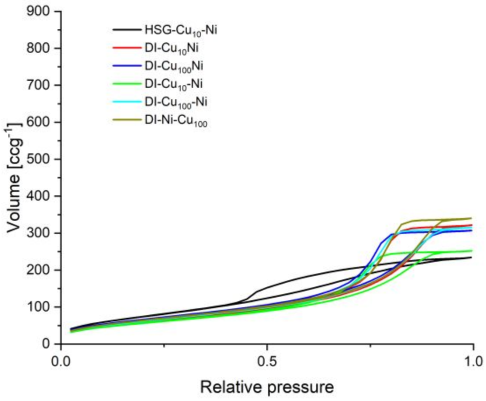

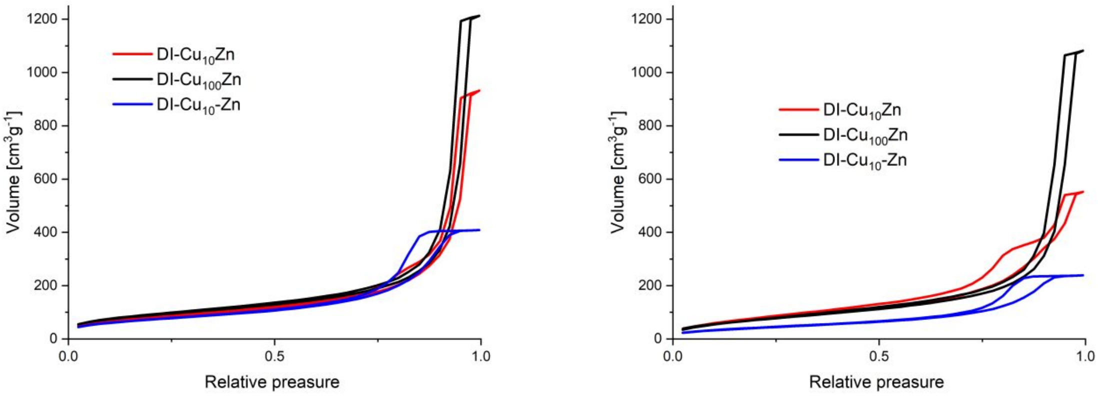

Figure S11. *Graph: N_2 isotherms for Ni and Zn doped catalysts. The left graph is for fresh-calcined samples before catalysis. Spent samples after the whole catalytic test measurement (right).*

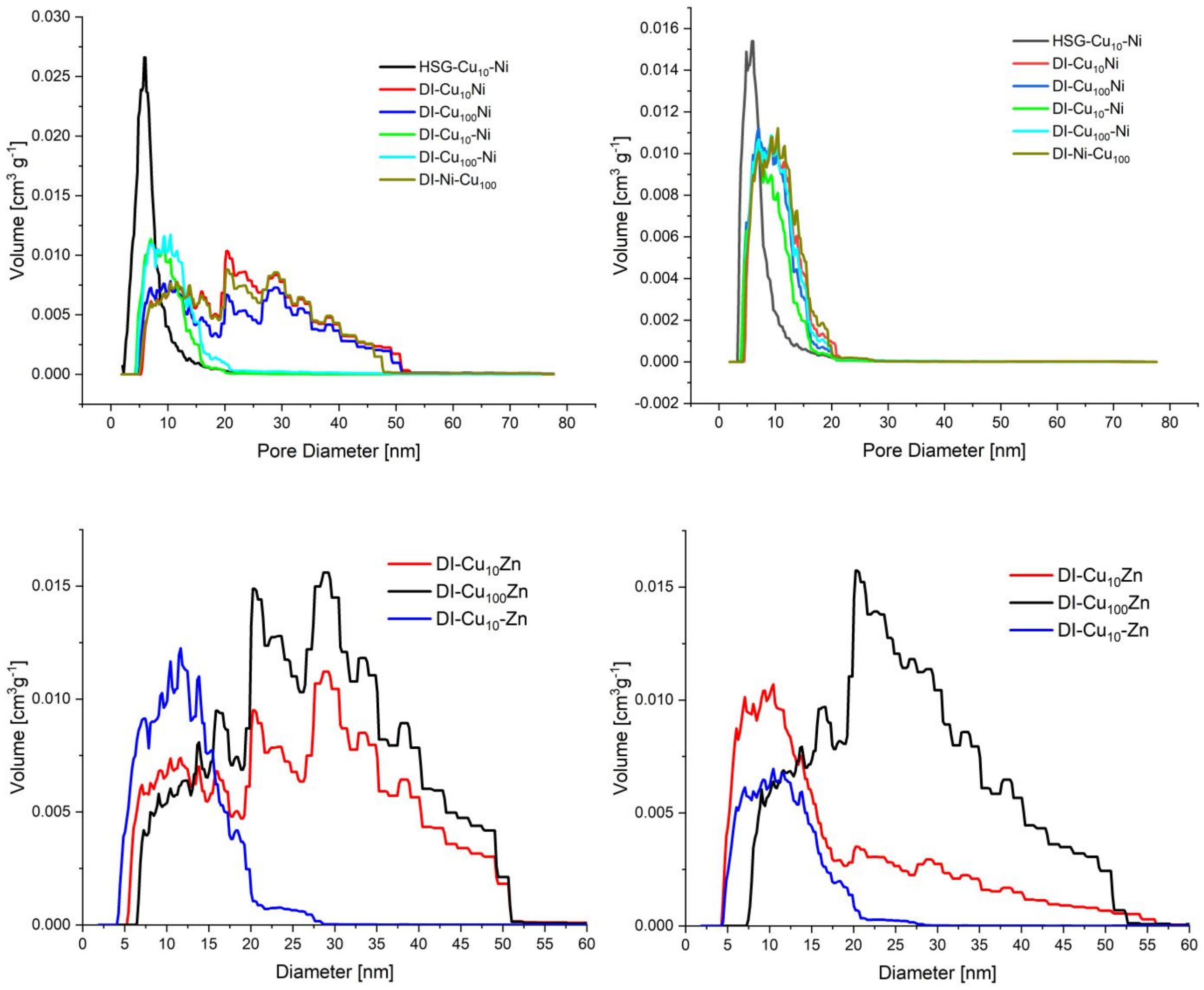

Figure S12. *Comparison of pore size distribution in CuNi catalysts (left fresh-calcined, right spent)*

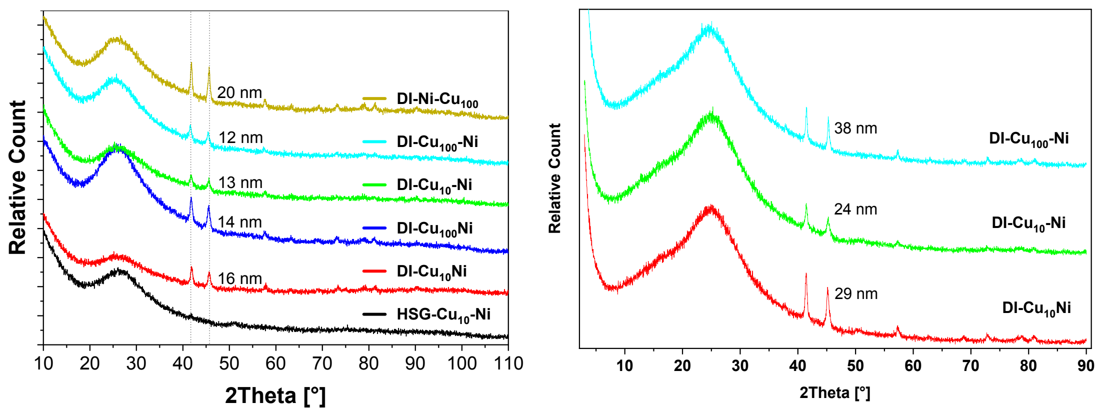

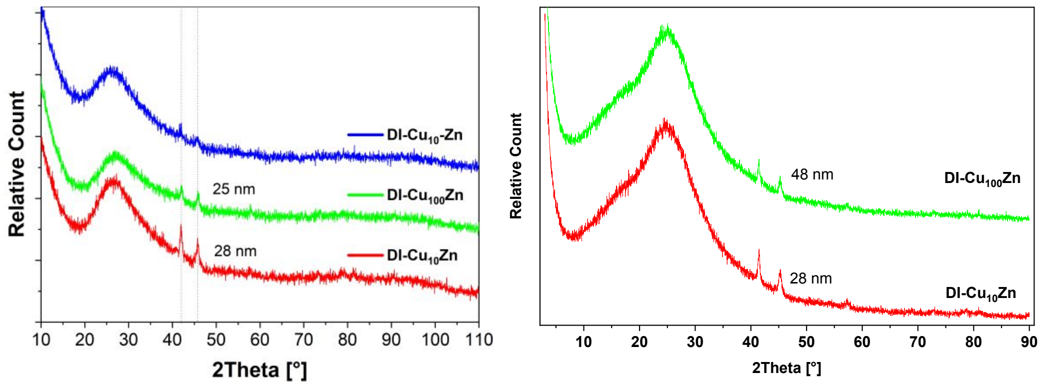

Figure S13. *PXRD patterns of Ni-doped (up) and Zn-doped (down) fresh catalysts (calcined in air) with Debye-Scherrer estimation of crystallite size. Selected samples were re-analyzed applying a lower scanning rate and step to obtain a better S/N ratio (diffractograms on the right side).*

Table S2. *CuO lattice parameters estimated for the parent sample DI-Cu and for the Ni-doped samples re-analysed with a lower scanning rate and step.*

**Sample**	**d (Å)***	**Lattice parameters (Å)**		
		**a**	**b**	**c**
CuO**	2.5324	4.6883	3.4229	5.1319
DI-Cu	2.5243	4.6797	3.4290	5.1374
DI-Cu_{10}Ni	2.5261	4.6894	3.4287	5.1360
DI-Cu_{10}-Ni	2.5270	4.6887	3.4282	5.1353
DI-Cu100-Ni	2.5266	4.6841	3.4294	5.1306

*d reported for the most intense diffraction (002 at 41.5°); **98-003-1059

Table S3. *CuO lattice parameters estimated for the parent sample DI-Cu and for the Zn-doped samples re-analysed with a lower scanning rate and step.*

Sample	d (Å)*	Lattice parameters (Å)		
		a	b	c
CuO**	2.5324	4.6883	3.4229	5.1319
DI-Cu	2.5243	4.6797	3.4290	5.1374
DI-Cu_{100}Zn	2.5270	4.6806	3.4321	5.1372
DI-Cu_{10}Zn	2.5272	4.6910	3.4302	5.1367

*d reported for the most intense diffraction (002 at 41.5°); **98-003-1059

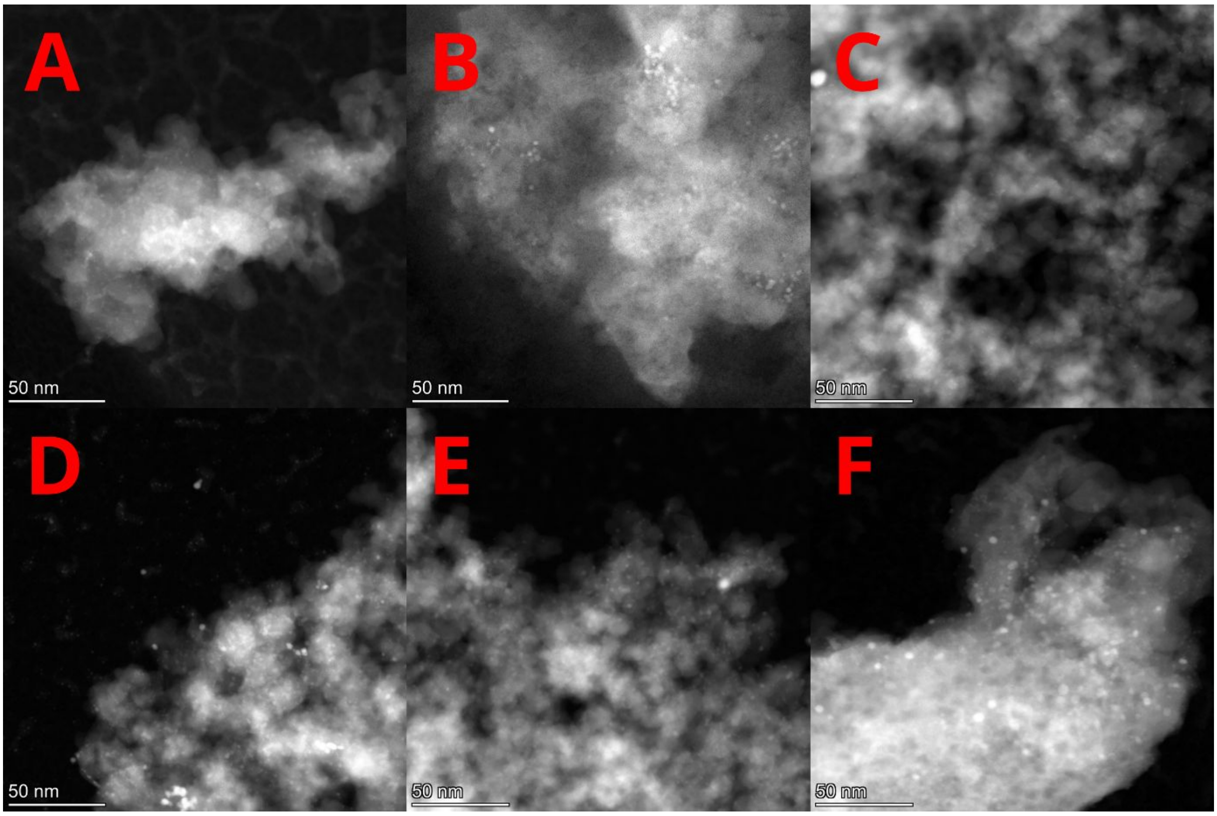

Figure S14. *STEM micrograph survey of Ni-doped fresh-reduced catalysts (calcined in air followed by hydrogen pretreatment; A:* ***HSG-Cu_{10}-Ni****, B:* ***DI-Cu_{10}Ni****, C:* ***DI-Cu_{100}Ni****, D:* ***DI-Cu_{10}-Ni****, E:* ***DI-Cu_{100}-Ni****, and F:* ***DI-Ni-Cu_{100}****.)*

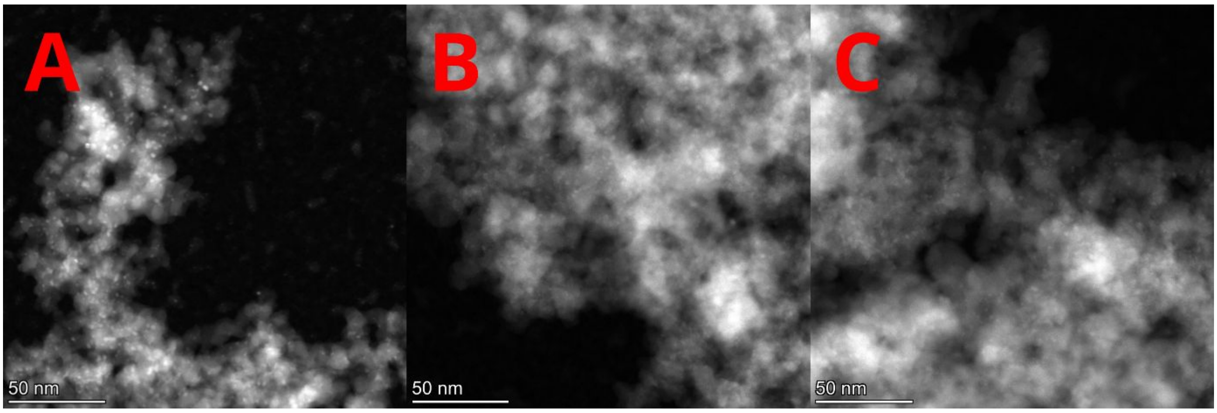

Figure S15. *STEM micrograph survey of Zn-doped fresh-reduced catalysts (calcined in air followed by hydrogen pretreatment; A:* ***DI-Cu$_{10}$Zn*** *B:* ***DI-Cu$_{100}$Zn****, C:* ***DI-Cu$_{10}$Zn****)*

A

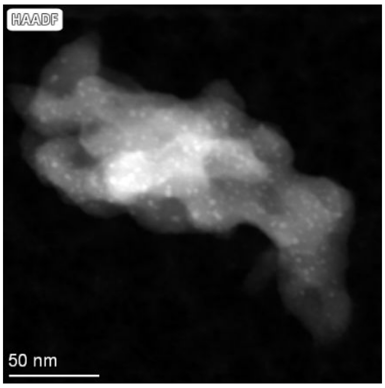

B

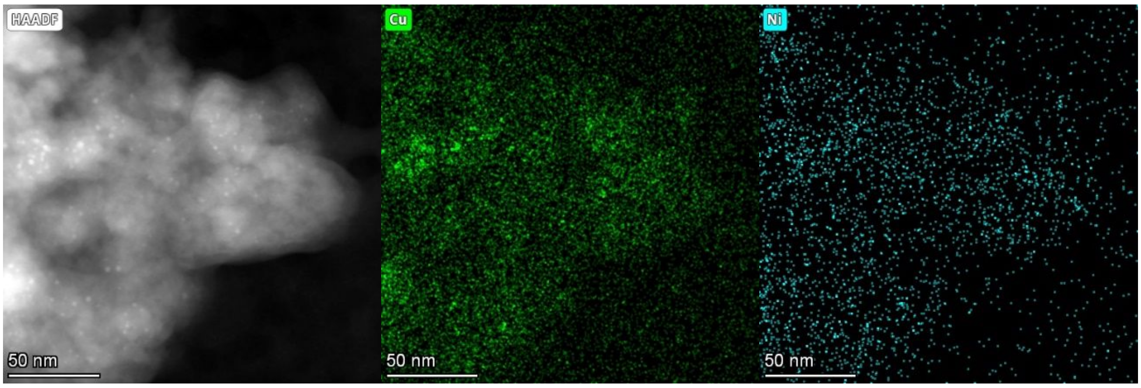

C

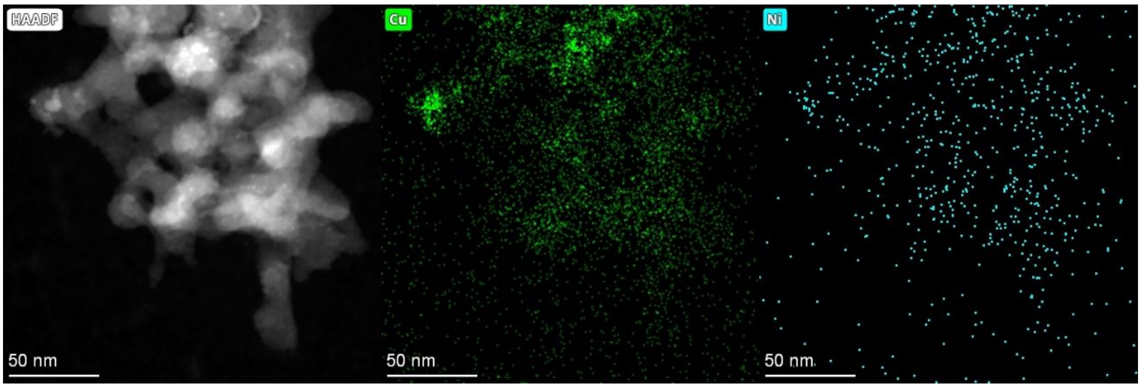

HAADF
50 nm
Cu
50 nm
Ni
50 nm

D

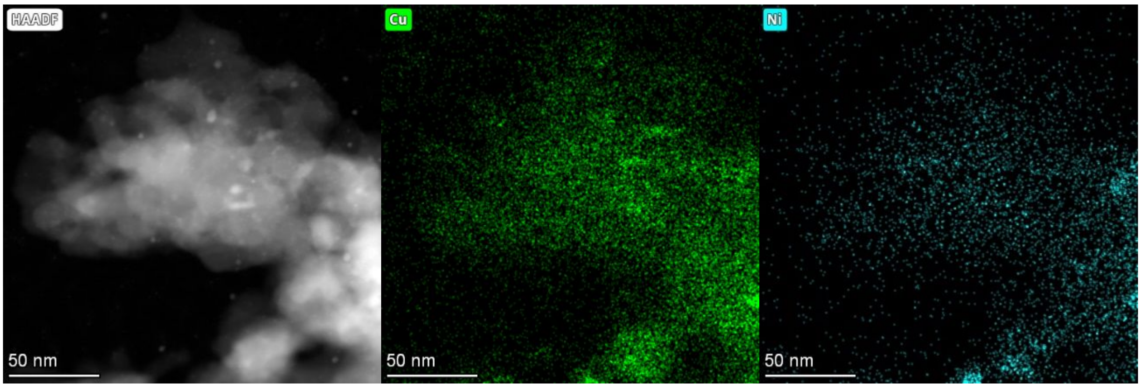

HAADF
50 nm
Cu
50 nm
Ni
50 nm

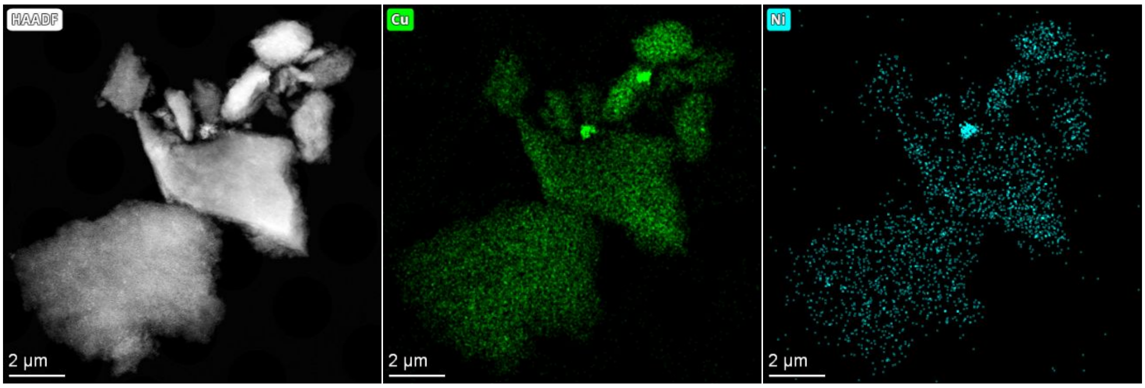

HAADF
2 µm
Cu
2 µm
Ni
2 µm

E

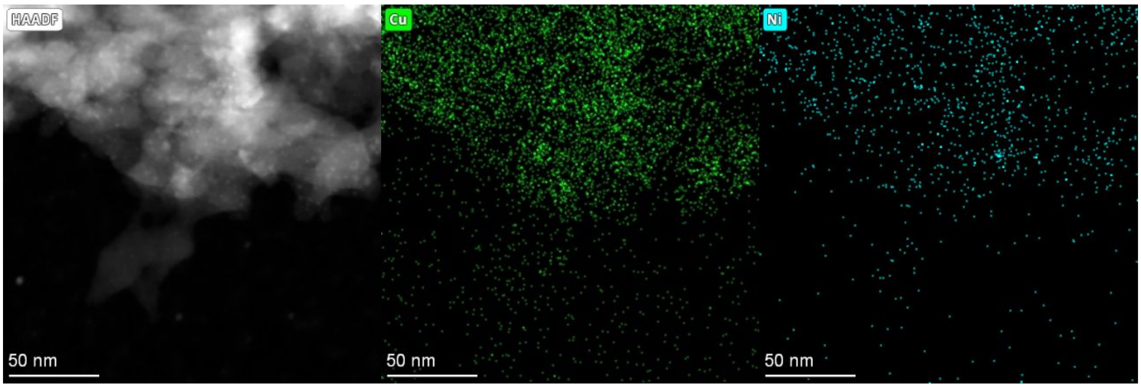

F

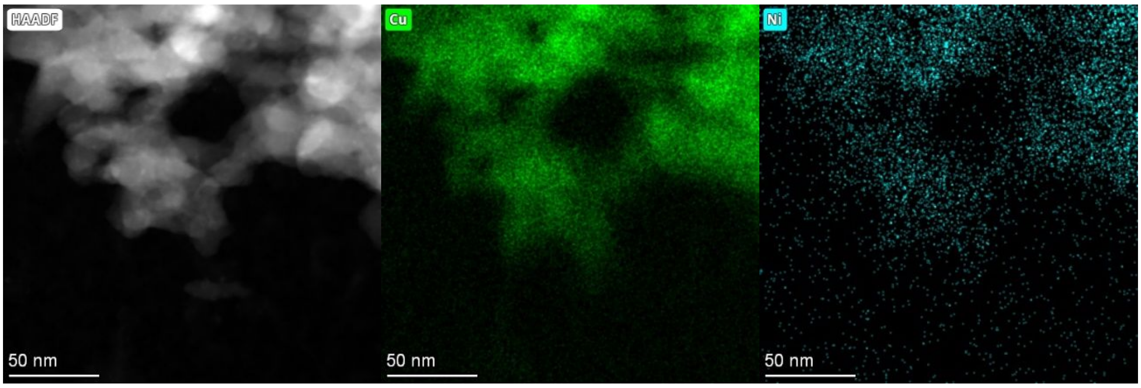

Figure S16. *STEM-EDS micrograph survey and elemental mapping of Ni-doped fresh-reduced catalysts (calcined in air followed by hydrogen pretreatment; A:* ***HSG-Cu$_{10}$-Ni****, B:* ***DI-Cu$_{10}$Ni****, C:* ***DI-Cu$_{100}$Ni****, D:* ***DI-Cu$_{10}$-Ni****, E:* ***DI-Cu$_{100}$-Ni****, and F:* ***DI-Ni-Cu$_{100}$****.)*

A

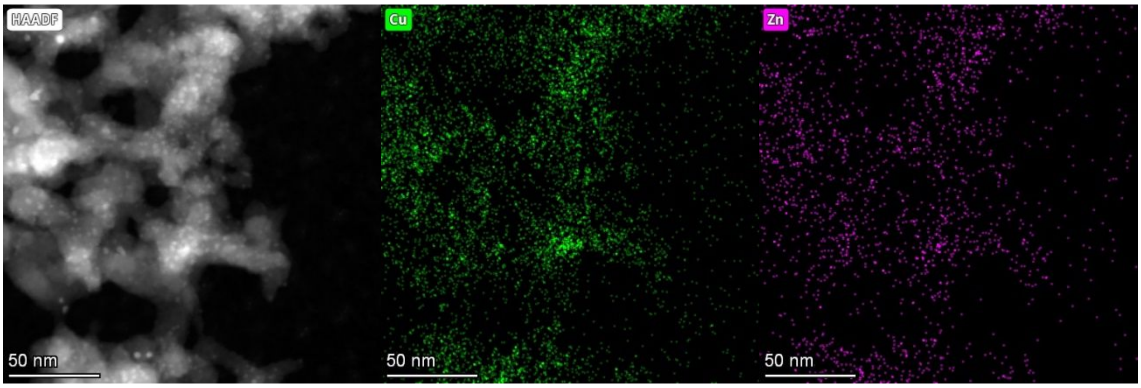

B

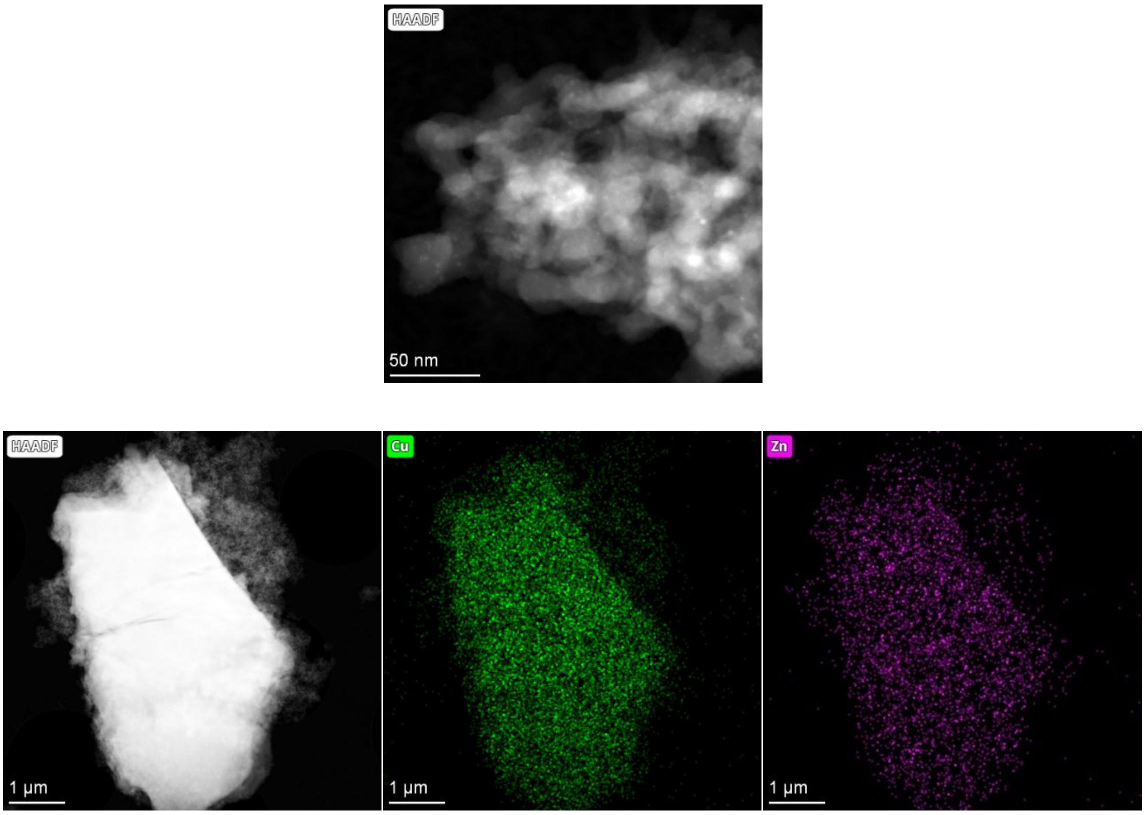

C

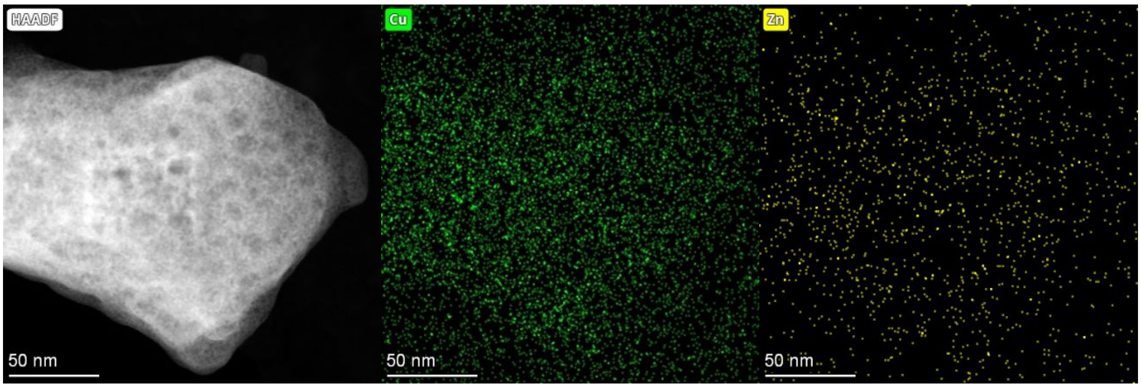

Figure S17. *STEM-EDS micrograph survey and elemental mapping of Zn-doped fresh-reduced catalysts (calcined in air followed by hydrogen pretreatment; A:* ***DI-Cu$_{10}$Zn*** *B:* ***DI-Cu$_{100}$Zn****, C:* ***DI-Cu$_{10}$-Zn****)*

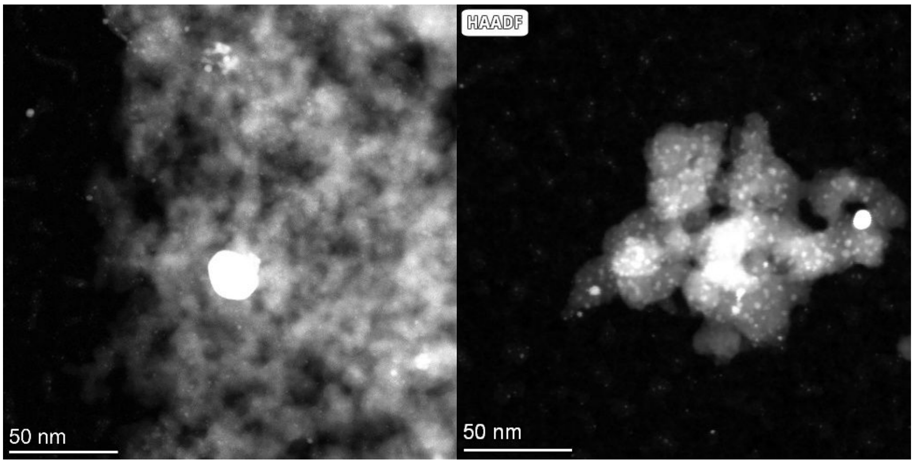

Figure S18. *Inhomogeneous copper distribution observed by STEM corelating with PXRD analyses (samples* ***DI-Cu$_{10}$-Ni*** *and* ***DI-Cu$_{10}$Zn****, left and right, respectively).*

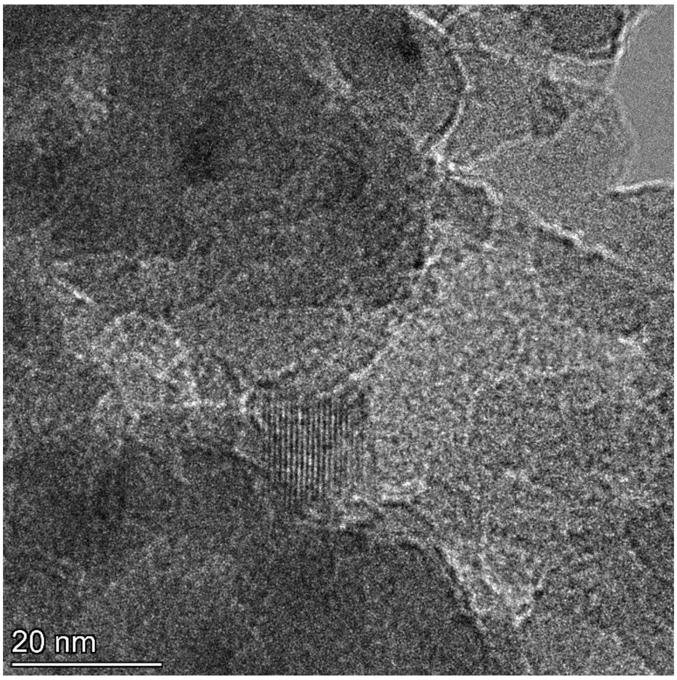

Figure S19*: STEM micrograph of sample* ***DI-Cu10-Ni*** *showing a crystalline Ni-rich particle with interplanar spacing ~2.1Å.*

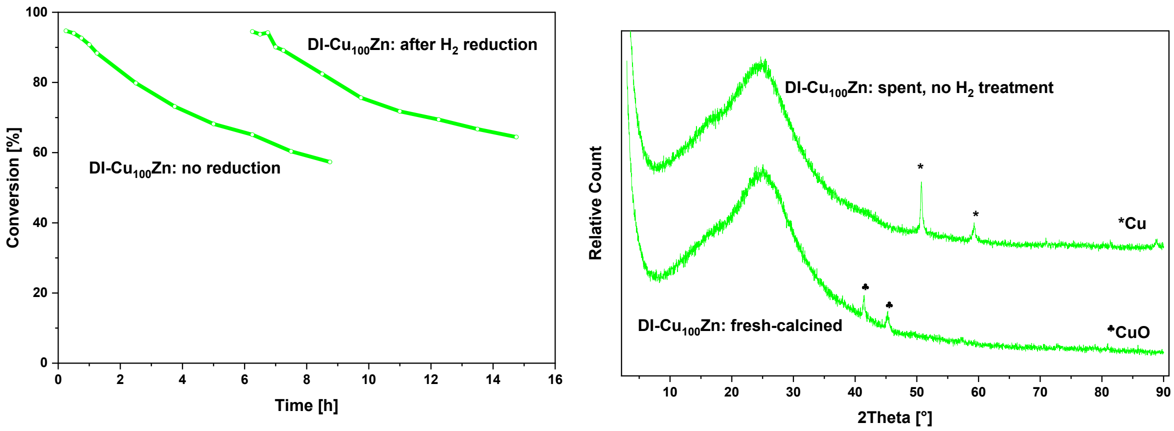

***Figure S20**: Comparison of catalytic performance of fresh-calcined and fresh-reduced **DI-Cu$_{100}$Zn** (left side). The difference in time is given by the H_2-pretreatment and temperature ramping performed in the case of fresh-reduced catalysts before reaching 325 °C. XRD diffractograms of fresh-calcined and spent DI-Cu$_{100}$Zn (right side).*

***Table S4.** Average selectivity to acetaldehyde for different catalysts during the catalytic experiments*

Sample	Selectivity to acetaldehyde 185 °C [%]	Selectivity to acetaldehyde 220 °C [%]	Selectivity to acetaldehyde 255 °C [%]	Selectivity to acetaldehyde 290 °C [%]
**HSG-Cu$_{10}$-Ni**	90	88	89	95
**DI-Cu$_{10}$Ni**	67	69	63	66
**DI-Cu$_{100}$Ni**	64	71	76	77
**DI-Cu$_{10}$-Ni**	68	68	72	74
**DI-Cu$_{100}$-Ni**	98	91	90	92
**DI-Ni-Cu$_{100}$**	70	87	90	91
**DI-Cu$_{10}$Zn**	75	88	91	92
**DI-Cu$_{100}$Zn**	85	91	91	92
**DI-Cu$_{10}$-Zn**	64	85	89	90

Table S5. *Average carbon balance for different catalysts during the catalytic experiments at 325 °C*

Sample	Carbon balance [%]	Standard deviation [%]
**HSG-Cu10-Ni**	99.98	3.09
**DI-Cu10Ni**	99.41	5.46
**DI-Cu100Ni**	99.54	0.99
**DI-Cu10-Ni**	96.85	1.87
**DI-Cu100-Ni**	96.85	2.89
**DI-Ni-Cu100**	97.37	0.85
**DI-Cu10Zn**	98.34	1.08
**DI-Cu100Zn**	98.22	2.79
**DI-Cu10-Zn**	99.11	1.00

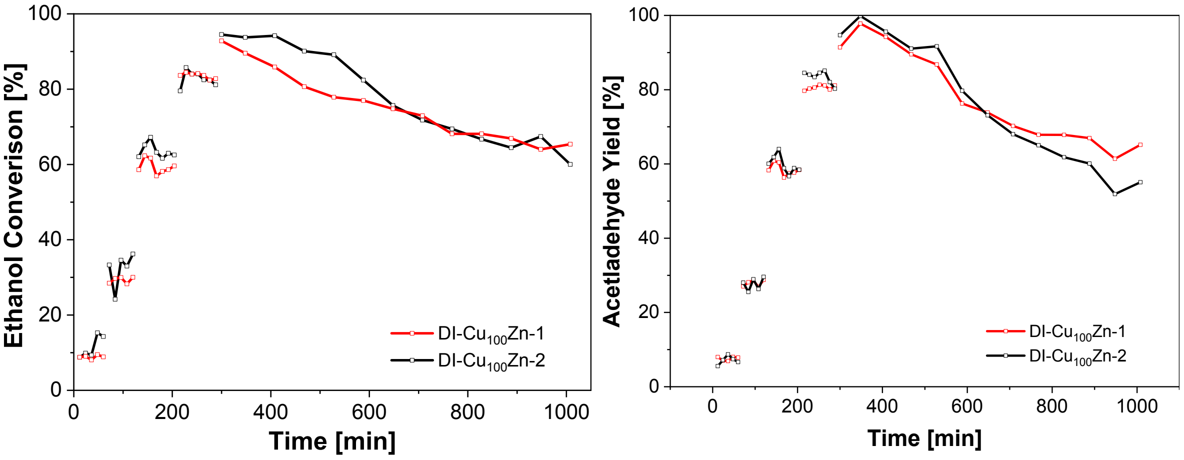

Figure S21. *Reproducibility of ethanol conversion and acetaldehyde yield over* ***DI-Cu$_{100}$Zn sample****. Each step represents one temperature (185, 220, 255, 290 °C, and 325 °C). Reaction conditions: 100 mg of catalyst, 50 ml min^{-1} N_2, 4.73 g g^{-1} h^{-1} of ethanol.*

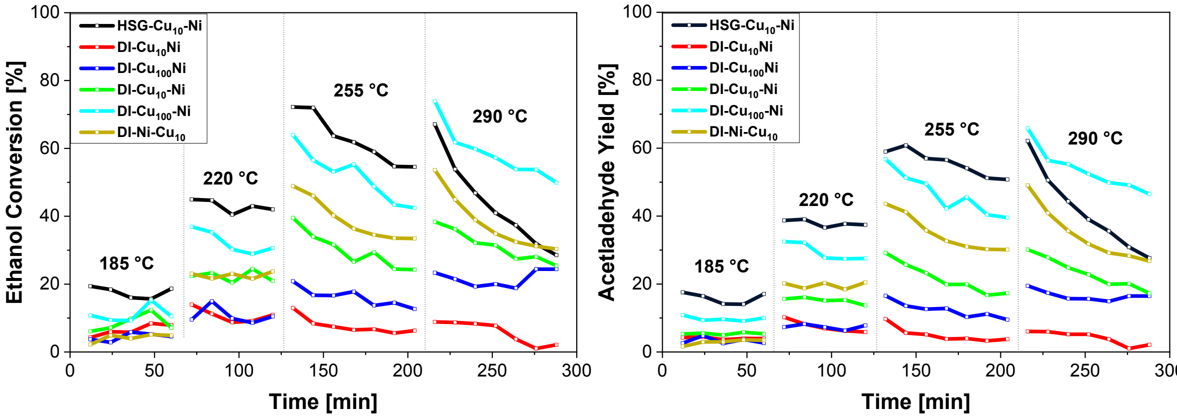

Figure S22. *Comparison of ethanol conversion and acetaldehyde yield over the Ni-doped catalysts. Each step represents one temperature (185, 220, 255 and 290 °C). Reaction conditions: 100 mg of catalyst, 50 ml min^{-1} N_2, 4.73 g g^{-1} h^{-1} of ethanol.*

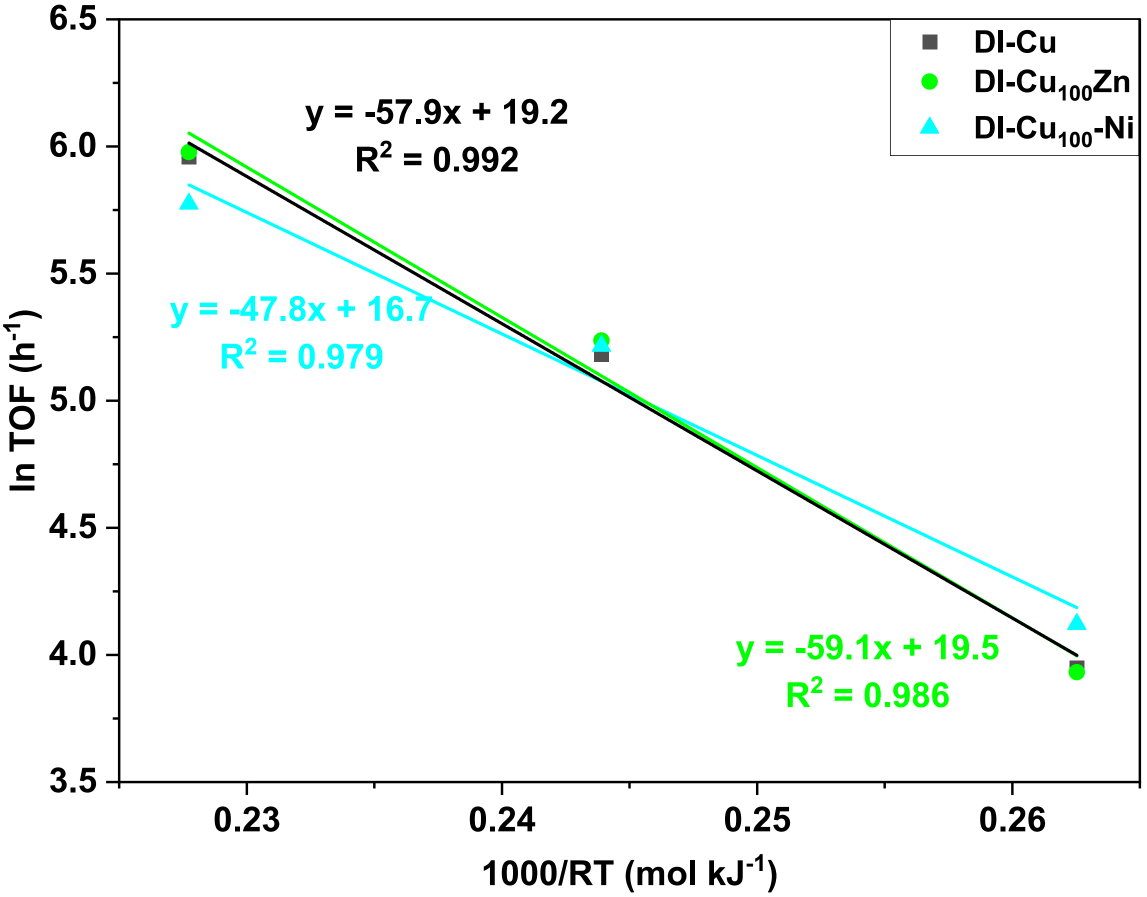

Figure S23. *Arrhenius plots for DI-Cu, DI-Cu$_{100}$Zn, and DI-Cu$_{100}$-Ni catalysts.*

***Table S6**. Parameters used for the estimation of apparent activation energy (E_a).*

Sample	d_{NPs} (nm)	Cu_{bulk} (wt%)	Cu_{surf} (mmol g_{CATA}^{-1})	T (°C)	TOF ($mol_{acetaldehyde}$ mol_{Cu}^{-1} h^{-1})	E_a (kJ mol^{-1})
**DI-Cu**	2.5	2.42	0.158	185	51.9	58±5
				220	178	
				255	387	
**DI-Cu_{100}Zn**	2.8	2.61	0.152	185	51.0	59±7
				220	188	
				255	395	
**DI-Cu_{100}-Ni**	2.1	2.33	0.181	185	61.7	48±7
				220	184	
				255	322	

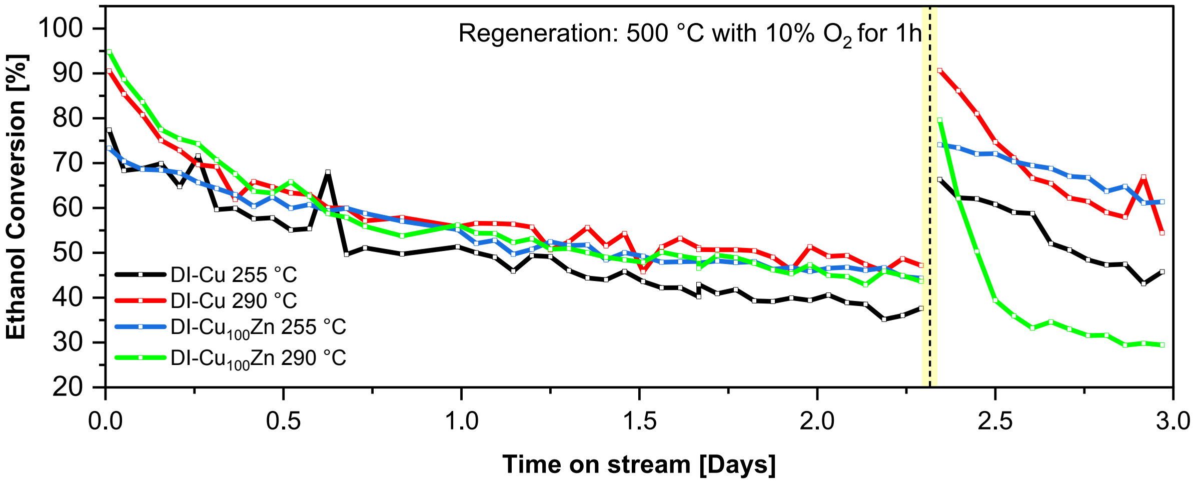

Figure S24. Long-term stability test of **DI-Cu_{100}Zn** and parent DI catalyst at 255 °C and 290 °C with following reaction conditions: 100 mg of catalyst, 50 ml min^{-1} N_2, 4.73 g g^{-1} h^{-1} of ethanol (regeneration: 10% O_2 in N_2, 500 °C for 1 hour).

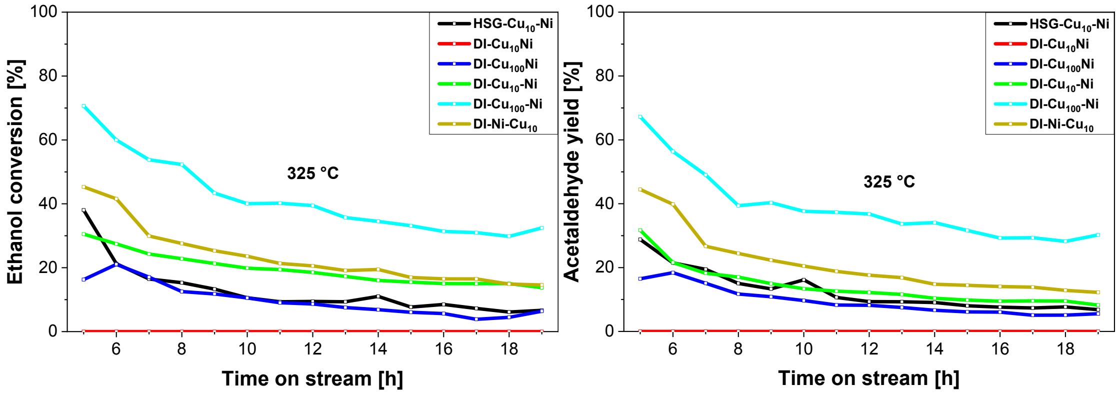

Figure S25. *High temperature (325 °C) stability test of Ni-doped catalysts. Ethanol conversion and acetaldehyde yield analysed at reaction conditions: 100 mg of catalyst, 50 ml/min N_2, 4.73 g g^{-1} h^{-1} of ethanol.*

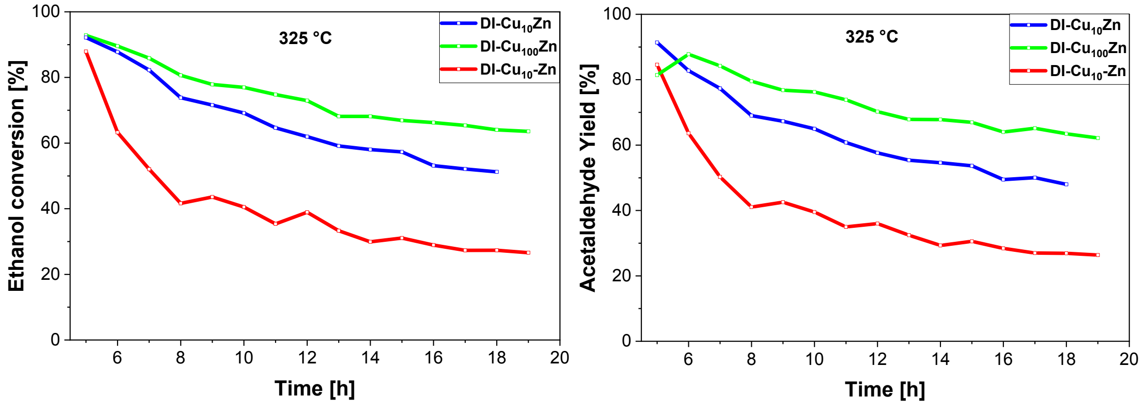

Figure S26. *High temperature (325 °C) stability test of Zn-doped catalysts. Ethanol conversion and acetaldehyde yield analysed at reaction conditions: 100 mg of catalyst, 50 ml/min N_2, 4.73 g g^{-1} h^{-1} of ethanol.*

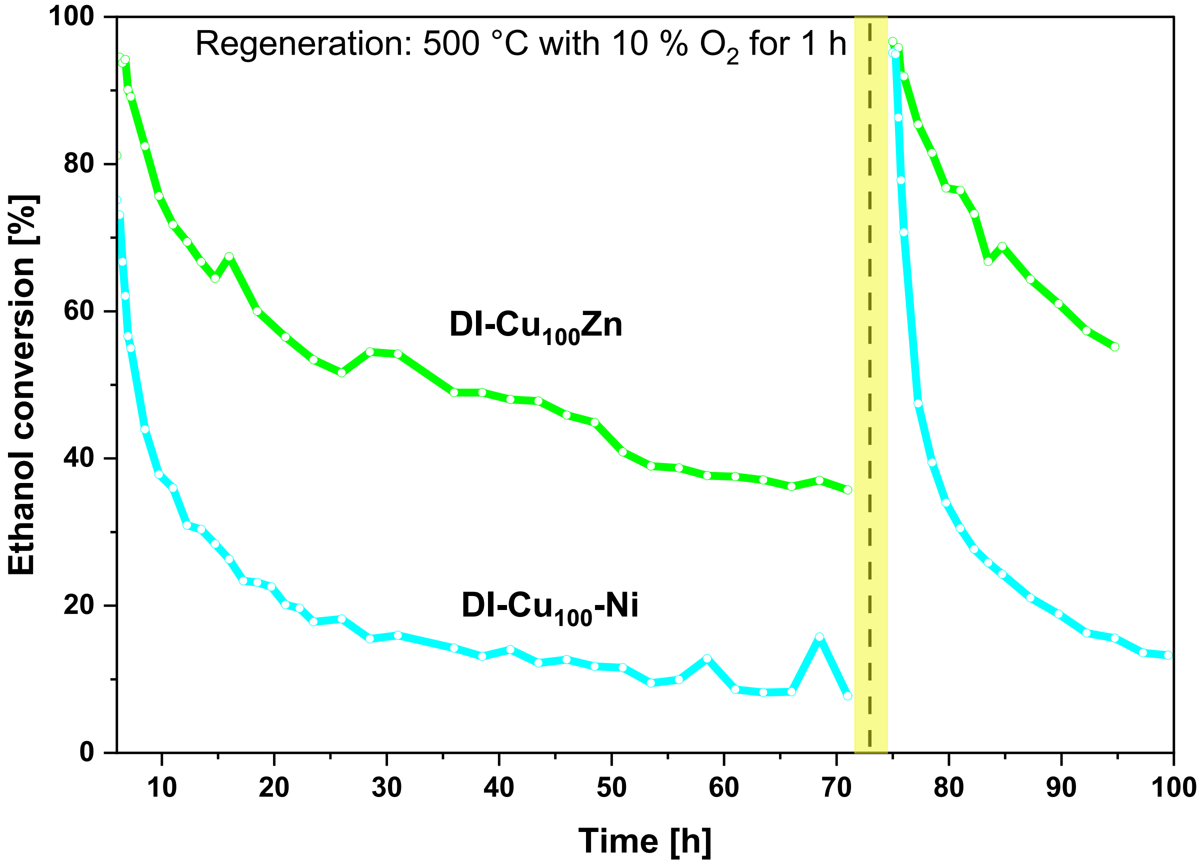

__Figure S27.__ Long-term high temperature (325 °C) stability test of __DI-Cu$_{100}$Zn__ and __DI-Cu$_{100}$-Ni__ catalysts. Ethanol conversion and acetaldehyde yield analysed at reaction conditions: 100 mg of catalyst, 50 ml/min N_2, 4.73 g g^{-1} h^{-1} of ethanol.

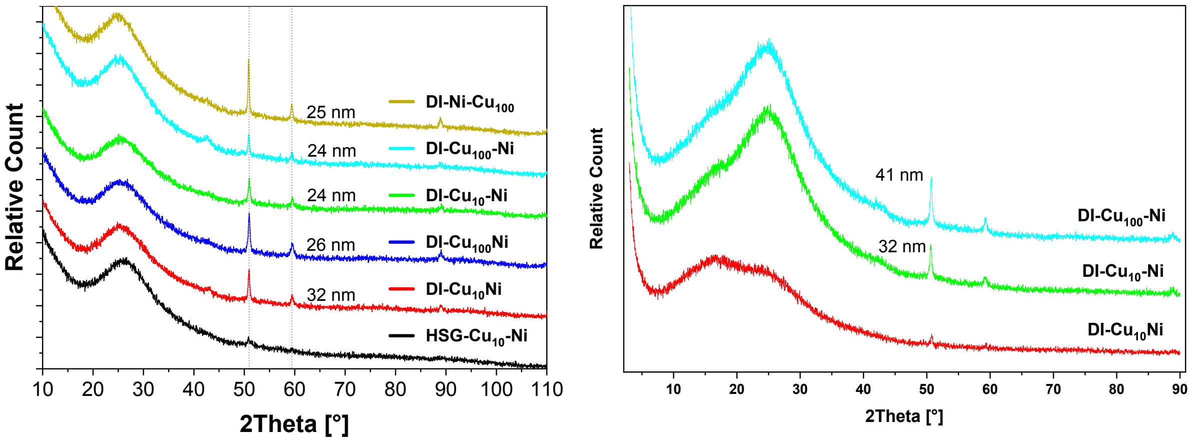

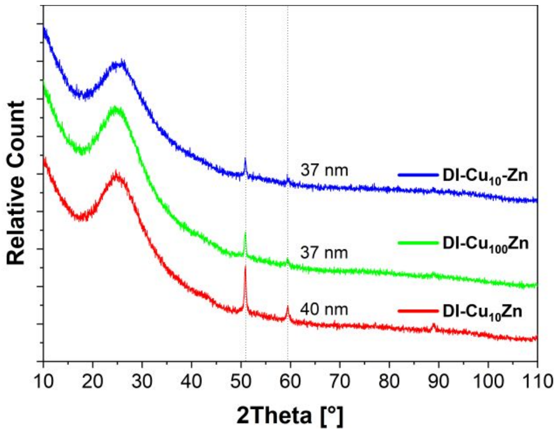

Figure S28. *PXRD patterns of spent Ni-doped (up) and Zn-doped (down) catalysts with crystallite sizes estimated by Debye-Scherrer equation. Selected Ni-doped samples were re-analyzed applying a lower scanning rate and step to obtain a better S/N ratio (diffractogram on the right side)*

Table S7. *CuO lattice parameters estimated for the spent parent sample DI-Cu and for the spent Ni-doped samples re-analysed with a lower scanning rate and step.*

**Sample**	**d (Å)***	**Lattice parameter a (Å)**
Cu**	2.0880	3.6150
Ni***	2.0340	3.5238
DI-Cu	2.0874	3.6157
DI-Cu_{10}Ni	2.0880	3.6183
DI-Cu_{10}-Ni	2.0943	3.6261
DI-Cu_{100}-Ni	2.0915	3.6206

*d reported for the most intense diffraction (111 at 50.7°); **98-062-7113; ***87-712

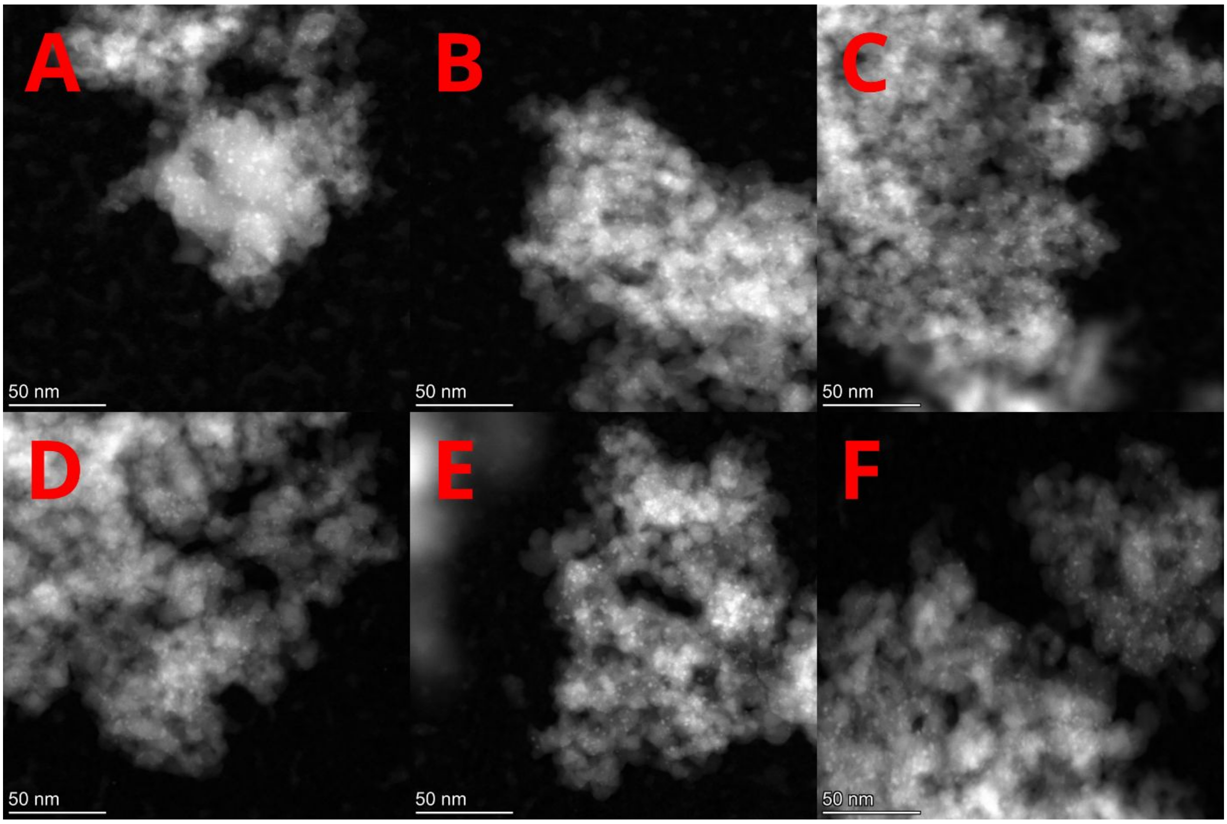

Figure S29. *STEM micrograph survey of Ni-doped spent catalysts (A:* ***HSG-Cu_{10}-Ni****, B:* ***DI-Cu_{10}Ni****, C:* ***DI-Cu_{100}Ni****, D:* ***DI-Cu_{10}-Ni****, E:* ***DI-Cu_{100}-Ni****, and F:* ***DI-Ni-Cu_{100}****.)*

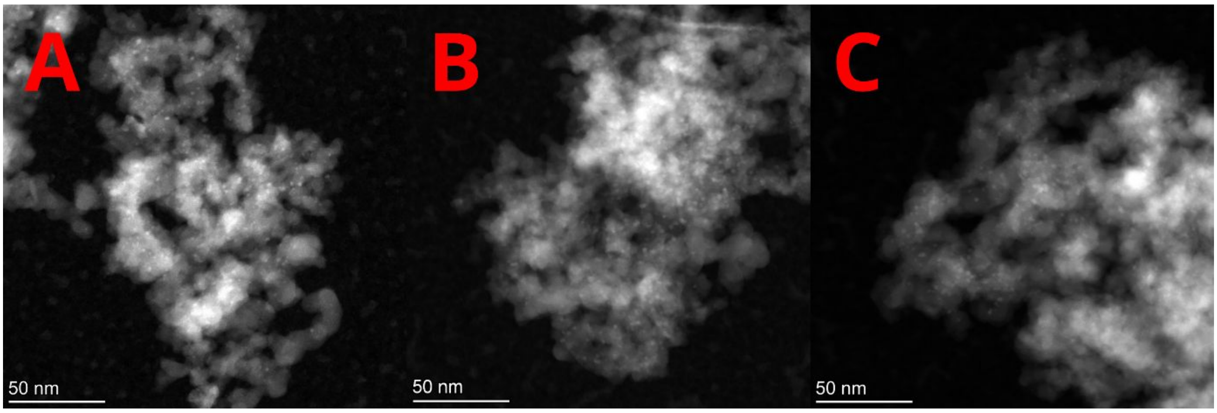

Figure S30. *STEM micrograph survey of Zn-doped spent catalysts (A:* ***DI-Cu_{10}Zn*** *B:* ***DI-Cu_{100}Zn****, C:* ***DI-Cu_{10}-Zn****)*

A

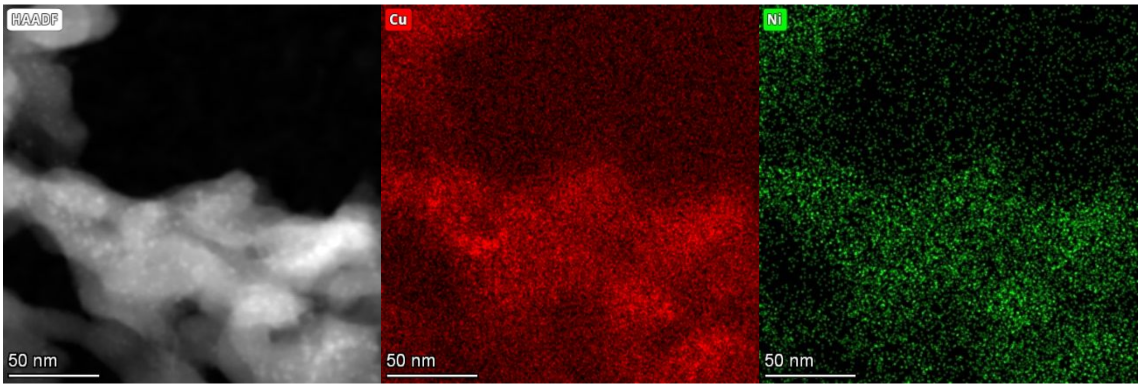
HAADF
50 nm
Cu
50 nm
Ni
50 nm

B

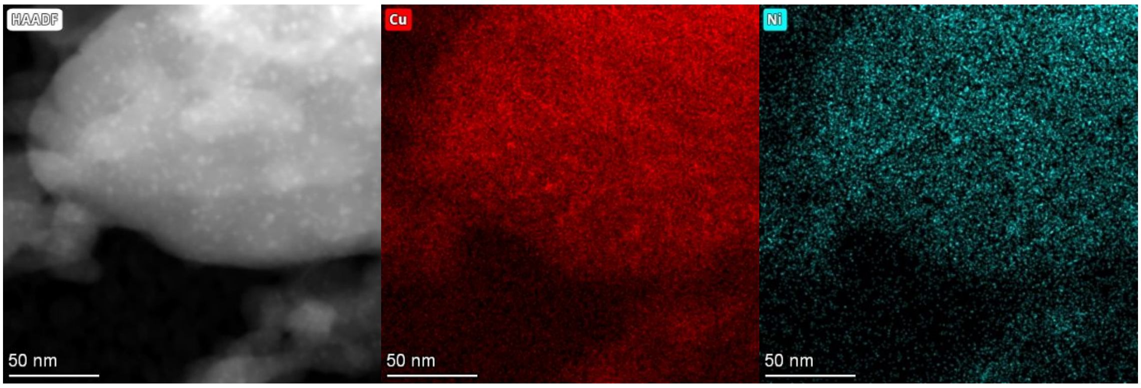
HAADF
50 nm
Cu
50 nm
Ni
50 nm

C

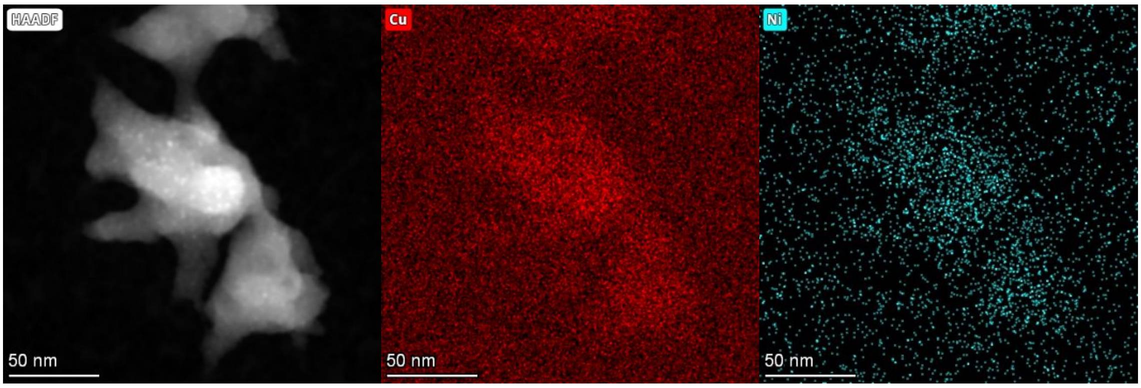
HAADF
50 nm
Cu
50 nm
Ni
50 nm

D

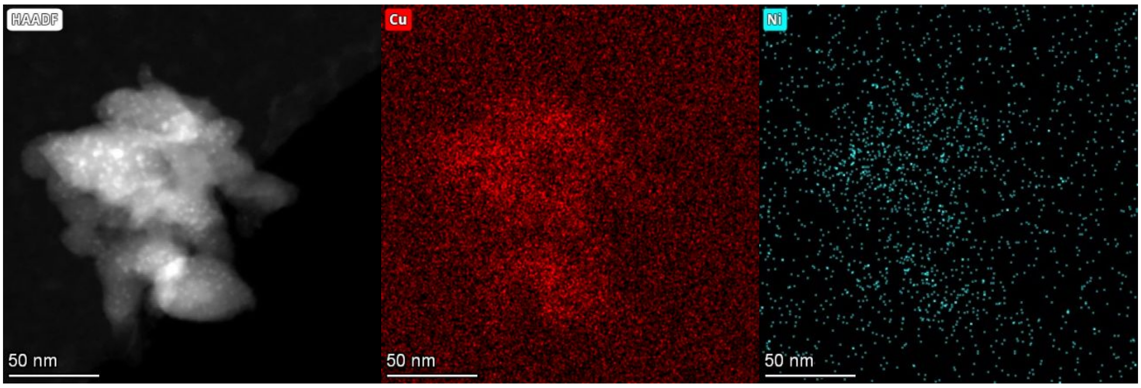

E

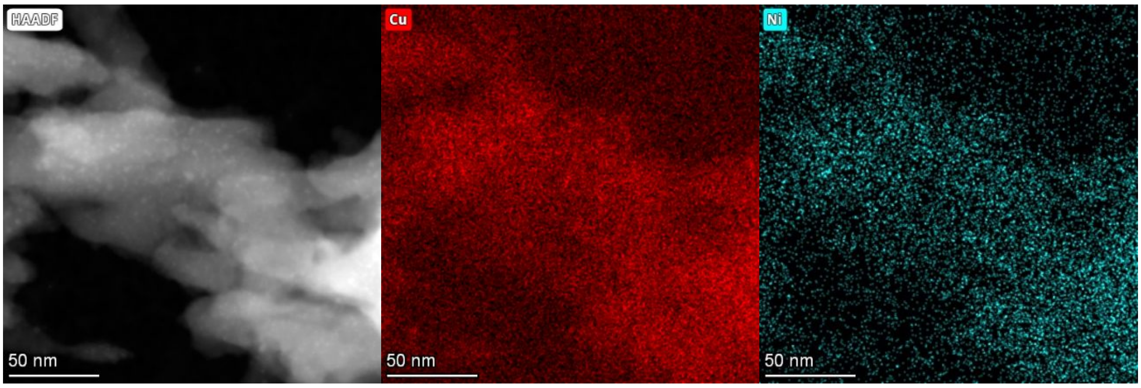

F

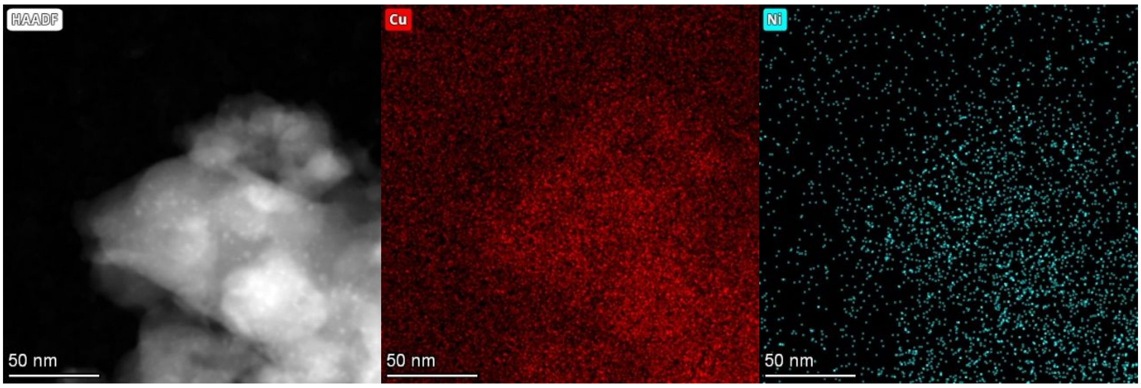

Figure S31. *STEM-EDS micrograph survey and elemental mapping of Ni-doped spent catalysts (A: **HSG-*Cu_{10}*-Ni**, B: **DI-*Cu_{10}*Ni**, C: **DI-*Cu_{100}*Ni**, D: **DI-*Cu_{10}*-Ni**, E: **DI-*Cu_{100}*-Ni**, and F: **DI-Ni-*Cu_{100}*.)*

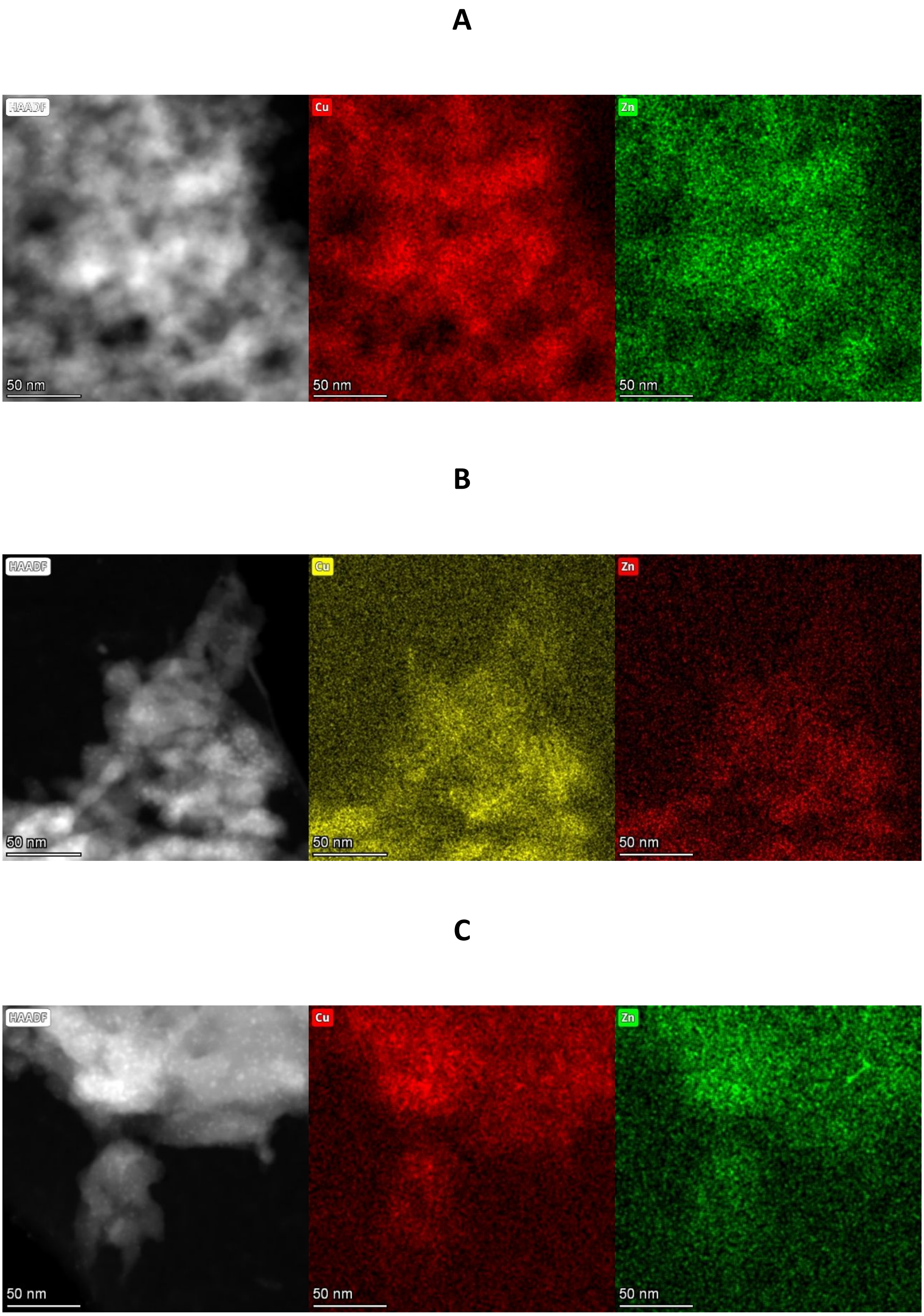

Figure S32. *STEM-EDS micrograph survey and elemental mapping of Zn-doped spent catalysts (A:* ***DI-Cu_{10}Zn*** *B:* ***DI-Cu_{100}Zn****, C:* ***DI-Cu_{10}-Zn****)*

Table S8. *Cu/Si ratios based on Cu and Si wt% determined from XPS analyses in fresh-calcined and spent catalysts.*

Sample	Cu/Si ratio [-]	
	Fresh	Spent
**HSG-Cu$_{10}$-Ni**	0.0156	0.0073
**DI-Cu$_{10}$Ni**	0.0022	0.0146
**DI-Cu$_{100}$Ni**	0.0017	0.0087
**DI-Cu-Ni$_{10}$**	0.0086	0.0072
**DI-Cu-Ni$_{100}$**	0.0067	0.0070
**DI-Ni$_{100}$-Cu**	0.0043	0.0070
**DI-Cu$_{10}$Zn**	0.0058	0.0079
**DI-Cu$_{100}$Zn**	0.0115	0.0112
**DI-Cu$_{10}$-Zn**	0.0111	0.0111

References

(1) Wang, Z.; Liu, Q.; Yu, J.; Wu, T.; Wang, G. Surface Structure and Catalytic Behavior of Silica-Supported Copper Catalysts Prepared by Impregnation and Sol–Gel Methods. *Appl. Catal. A Gen.* **2003**, *239* (1–2), 87–94. https://doi.org/10.1016/S0926-860X(02)00421-0.

(2) van den Berg, R.; Parmentier, T. E.; Elkjær, C. F.; Gommes, C. J.; Sehested, J.; Helveg, S.; de Jongh, P. E.; de Jong, K. P. Support Functionalization To Retard Ostwald Ripening in Copper Methanol Synthesis Catalysts. *ACS Catal.* **2015**, *5* (7), 4439–4448. https://doi.org/10.1021/acscatal.5b00833.
